# Microglial Feimin Alleviates Cognitive Impairment in High‐Fat Diet‐Fed Mice

**DOI:** 10.1002/advs.202512023

**Published:** 2025-10-20

**Authors:** Ran Gao, Zhonghua Xiong, Wenting Su, Jiahui Deng, Bin Zhai, Gaizhi Zhu, Min Zhang, Qi Zeng, Jinming Qiu, Ziqing Bian, He Xiao, Guoming Luan, Renxi Wang

**Affiliations:** ^1^ Beijing Institute of Brain Disorders Laboratory of Brain Disorders Ministry of Science and Technology Collaborative Innovation Center for Brain Disorders Capital Medical University Beijing 100069 China; ^2^ Department of Neurosurgery SanBo Brain Hospital Capital Medical University Beijing 100093 China; ^3^ Headache Center Department of Neurology Beijing Tiantan Hospital Capital Medical University Beijing 100070 China; ^4^ Laboratory for Clinical Medicine Capital Medical University Beijing 100069 China; ^5^ Beijing Key Laboratory of Epilepsy Research Department of Brain Institute Center of Epilepsy Beijing Institute for Brain Disorders Sanbo Brain Hospital Capital Medical University Beijing 100069 China; ^6^ Department of Hematology The Second Medical Center & National Clinical Research Center for Geriatric Diseases Chinese PLA General Hospital Beijing 100853 China; ^7^ State Key Laboratory of Toxicology and Medical Countermeasures Beijing Institute of Pharmacology and Toxicology Beijing 100850 China

**Keywords:** Feimin, B230219D22Rik, BV2, Palmitic acid, High‐fat diets, Hippocampus, Lipid droplets, Neuroinflammation

## Abstract

Lipid droplet accumulation in microglia, microglia‐mediated neuroinflammation, and subsequent neuronal damage are hallmark features of high‐fat diet (HFD)‐induced cognitive impairment. In this analysis, this is proposed that a new molecule feimin (B230219D22Rik in mice) is a key negative regulator of LD accumulation and the inflammatory response in HFD‐induced cognitive impairment. To test this hypothesis, BV2 microglia is exposed to palmitic acid (PA) in vitro, mimicking the effects of an HFD. This is found that feimin expression is significantly increased following high‐lipid stimulation. Feimin‐specific knockdown in BV2 cells led to enhanced LD accumulation, exacerbated inflammatory responses and neuronal apoptosis, whereas feimin overexpression has the opposite effect. Mechanistically, immunoprecipitation (IP) assays revealed that an interaction between feimin and AKT suppressed the AKT–mTOR signaling pathway. To further investigate the role of feimin in vivo, microglial feimin‐conditional knockout mice (feimin^Mic‐/−^) is developed. In the HFD model, feimin^Mic‐/−^ mice exhibited increased LD accumulation in hippocampal microglia, enhanced inflammation, and neuronal apoptosis, resulting in significant cognitive decline. In conclusion, this findings identified feimin as a key negative regulator of HFD‐induced LD accumulation and the microglia‐mediated inflammation response, suggesting that it is an attractive therapeutic target for cognitive decline associated with HFDs.

## Graphical abstract

Microglial feimin deletion exacerbates lipotoxicity‐induced neuroinflammation and cognitive decline. In both in vitro and in vivo models, loss of feimin aggravated lipid droplet accumulation, IL‐1β/IL‐6 release, and neuronal injury through the PI3K–AKT–mTOR pathway, ultimately leading to severe neuronal apoptosis and cognitive impairment under high‐fat diet conditions.

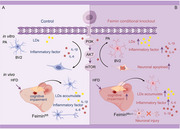

## 1 Introduction

Multiple systemic complications associated with high‐fat diets (HFDs) have a significant impact on the quality of life, including type 2 diabetes (T2D), cardiovascular disease, cancer, stroke, and Alzheimer's disease.^[^ ^1^, ^2^ ^]^ These health issues not only impose a substantial public health burden but also have profound social and economic consequences.^[^ ^3^ ^]^ Metabolic disturbances induced by HFDs lead to chronic inflammation and dysregulated lipid metabolism, resulting in hypertriglyceridemia, which adversely affects various organs and tissues, including the central nervous system (CNS).^[^ ^4^ ^]^ This condition substantially increases the risk of dementia and cognitive decline.^[^ ^5^ ^]^ Despite substantial epidemiological evidence supporting cognitive impairment as a common complication of HFD, it remains grossly underestimated.^[^ ^6^, ^7^, ^8^ ^]^

Neuroinflammation is a critical factor contributing to cognitive impairment and metabolic disease.^[^ ^9^ ^]^ Recent studies have highlighted the role of microglial activation in HFD‐induced cognitive deficits, particularly in brain regions essential for cognitive function, such as the hippocampus.^[^ ^10^, ^11^ ^]^ Chronic microglial activation and the excessive release of pro‐inflammatory mediators in the hippocampus lead to the expression of co‐stimulatory molecules, thereby initiating neuroinflammation and neuronal dysfunction.^[^ ^12^ ^]^ These neuroinflammatory responses result in neuronal apoptosis, reduced synaptic numbers, and impaired synaptic plasticity.^[^ ^13^ ^]^ These changes underscore the importance of microglia balance in determining the functional fate of neurons. The functional state of neurons directly influences the cognitive function of the hippocampus, which plays a central role in learning, memory, and other cognitive processes.^[^ ^14^, ^15^ ^]^

Lipid metabolism is a hallmark of HFD, with cells storing energy as neutral lipids in organelles known as lipid droplets (LDs) in response to nutrient fluctuations.^[^ ^16^ ^]^ LDs are critical in regulating inflammation, metabolic disorders, and cellular signaling, with a significant impact on microglia.^[^ ^16^ ^]^ In the diseased brain, microglial LDs organelles are not only lipid storage compartments, but also contribute to the onset and progression of neurodegenerative diseases by modulating oxidative stress, neuroinflammation, and energy metabolism.^[^ ^17^, ^18^ ^]^ LD regulates the release of inflammatory lipids, which, in turn, can influence the expression of inflammatory genes through PPAR or NF–κB signaling pathways.^[^ ^4^ ^]^ In HFD mice, the excessive accumulation of LD accelerates neurodegeneration, impairing learning and memory.^[^ ^19^ ^]^ Furthermore, genetic risk factors for Alzheimer's disease are closely linked to microglial LD accumulation.^[^ ^20^ ^]^ Studies have also reported the accumulation of LD in microglia and hippocampal cells in models of metabolic diseases, such as Type 2 Diabetes.^[^ ^21^, ^22^ ^]^ These findings further reinforce the relationship between microglial‐associated inflammation, LD accumulation, and cognitive decline.

Recent studies have reported that feimin is a skeletal muscle–derived secretory protein released upon feeding. It binds to the tyrosine kinase receptor MERTK and promotes glucose uptake while suppressing hepatic glucose production through the activation of the AKT signaling pathway. Feimin plays a critical role in regulating glucose homeostasis. It has been shown to significantly lower blood glucose levels in mouse models of obesity and diabetes, demonstrating a particularly synergistic effect when combined with insulin.^[^ ^23^ ^]^ In addition, recent research has shown that during exercise, cytoplasmic feimin in skeletal muscle interacts with FOXC2 during exercise, leading to the suppression of sarcolipin expression. This reaction, in turn, inhibits muscle thermogenesis and enhances exercise performance.^[^ ^24^ ^]^ This suggested that expression levels of feimin were increased in the plasma of diet‐induced obesity models, demonstrating the potential of this gene in the prevention and treatment of metabolic disorders. Our previous study reported that the expression of feimin was reduced in microglia in stroke models, and it has an inhibitory effect on inflammatory factors.^[^ ^25^ ^]^ However, the specific function of feimin in HFD‐associated microglia remains unknown. The present study highlights feimin as a key regulator of LD accumulation and microglial neuroinflammation induced by an HFD. We provide the first evidence that feimin expression is elevated in microglia in an HFD model and negatively regulates LD accumulation and inflammation by suppressing the AKT–mTOR pathway, correspondingly alleviating cognitive impairment in the in vivo HFD model.

## 2 Results

### 2.1 Feimin Alleviates PA‐Induced LD Accumulation and Inflammation in BV2 Cells

**Figure 1:**
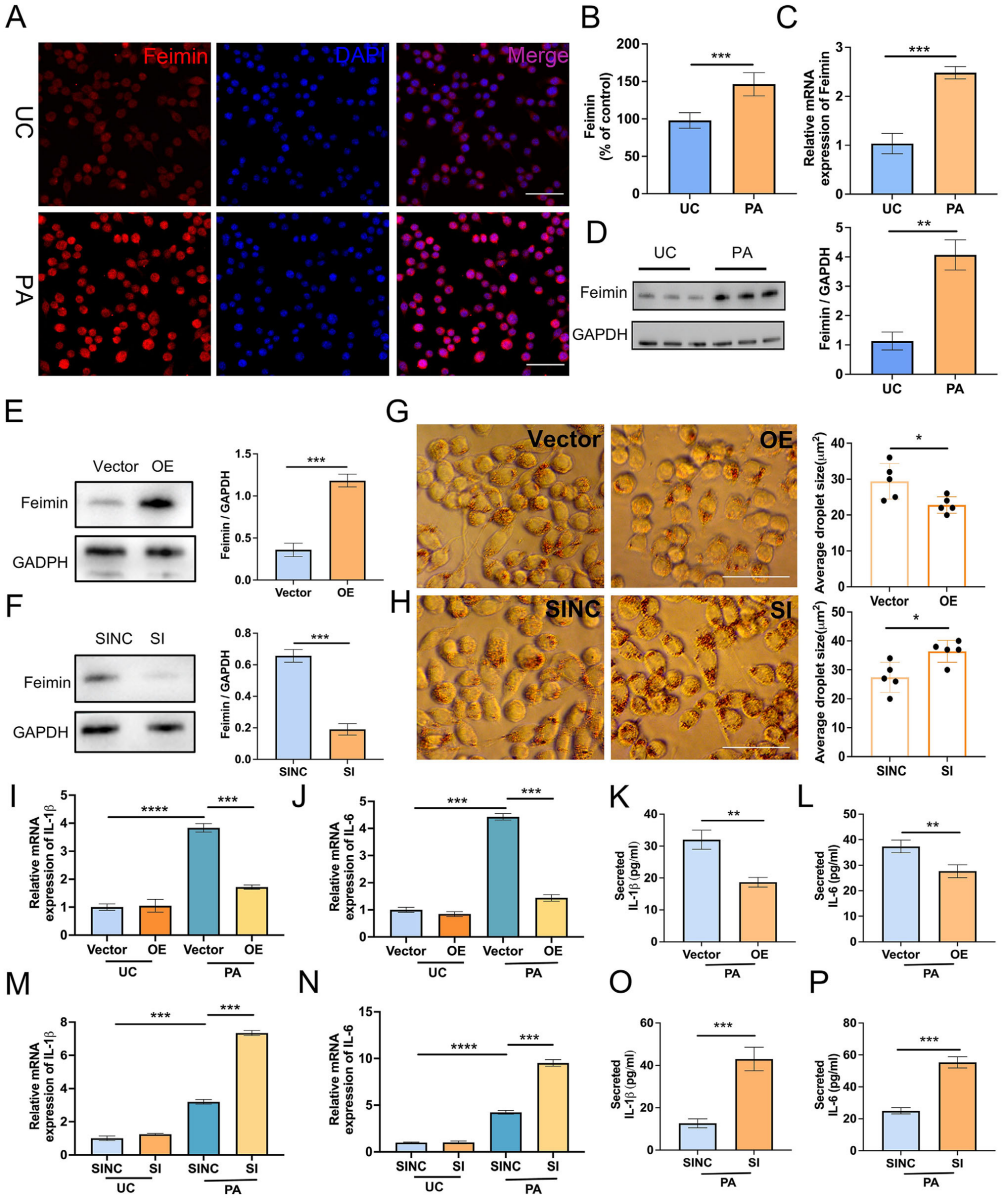
Effects of feimin on inflammation and lipid droplet (LD) accumulation in palmitic acid (PA)‐treated BV2 cells. A) Immunofluorescence (IF) staining and B) quantitative analysis of feimin expression in BV2 cells following 20 h PA treatment. Feimin is shown in red (anti‐feimin antibody staining) and nuclei are counterstained with blue (DAPI). N = 5, *p* = 0.0004, Scale bars: 50 µm. C,D) Feimin mRNA expression and protein levels were analyzed using qPCR assay and western blot (WB) in BV2 cells treated for 24 h with PA, n = 3, *p* = 0.0005 and 0.001. E,F) Feimin protein level were detected by western blot in BV2 cells with (E) feimin‐overexpression (OE) or (F) feimin‐specific siRNA (SI), n = 3, *p* = 0.0004 and 0.0003. G,H) LD distribution in BV2 cells was visualized using Oil Red O staining and LD area was measured with ImageJ in BV2 cells with feimin OE or feimin SI. N = 5, *p* = 0.027 and 0.013, Scale bars: 50 µm. LDs are stained red. I,J,M,N) The relative mRNA levels of IL‐1β and IL‐6 in BV2 cells with feimin OE (I,J, ****p < 0.0001, *p* = 0.0007,0.0005 and 0.0003) or feimin SI (M,N, ****p < 0.0001, *p* = 0.0006,0.0005 and 0.0002) were analyzed by qPCR, n = 3, ****p < 0.0001. K,L) The protein levels of IL‐1β and IL‐6 secreted in the culture medium of BV2 cells with feimin OE (*p* = 0.002, 0.009) or O,P) feimin SI were measured using ELISA, n = 5, *p* = 0.0009, 0.0002 (A–P) Data were presented as means ± SD of three independent experiments. OE: Feimin overexpression; SI: Small interfering RNA; SINC: siRNA negative control; UC: untreated control. Two‐group comparisons were analyzed by unpaired two‐tailed t‐tests (B–D, F–H, K,L, O,P) and multi‐group comparisons by one‐way ANOVA with multiple comparisons test (I,J, M,N). *p < 0.05, **p < 0.01, ***p < 0.001, ****p < 0.001.

According to previous studies,^[^ ^26^, ^27^, ^28^ ^]^ BV2 microglia‐induced LD accumulation triggers an inflammatory response, including the production of IL‐1β and IL‐6 in the palmitic acid (PA) model. To evaluate the role of feimin in a microglia‐mediated PA model, we first stimulated BV2 cells with PA for 24 h. We performed a dose–response assessment of PA on BV2 cells and found that concentrations up to 200 µM induced only mild cytotoxicity, with significant increases in inflammatory cytokine release and LD accumulation observed at 200 µM (Figure S1B–D, n = 3 in each group, Supporting Information). Consequently, 200 µM PA was selected for all subsequent experiments. Immunofluorescent (IF) staining, quantified using unbiased stereological methods to ensure accurate and representative sampling, revealed a significant increase in feimin expression following PA stimulation (**Figure** **1**A,B, *p* = 0.0004, n = 5 in each group). Consistently, qPCR confirmed feimin mRNA upregulation in PA‐treated cells (Figure 1C, *p* = 0.0005, n = 3 in each group), and western blot showed a parallel increase in feimin protein normalized to GAPDH (Figure 1D, *p* = 0.001, n = 3 in each group). To further investigate the function of feimin, we established a stable overexpression model (feimin OE) in BV2 cells via lentiviral transduction and verified successful overexpression (Figure 1E; Figure S1F—H, Supporting Information). In parallel, feimin expression was silenced using feimin‐SI and knockdown efficiency was validated (Figure 1F; Figure S1I, Supporting Information). Under physiological conditions, LDs did not accumulate in the vector control, feimin‐OE, siRNA negative control (SINC), or feimin siRNA (SI) groups (Figure S1A, n = 5, Supporting Information). We found that in the PA model, feimin OE exhibited significantly reduced LD accumulation (Figure 1G, 29.4 ± 5.0 and 22.8 ± 2.3 µm^2^, respectively, n = 5, *p* = 0.027). In contrast, feimin knockdown led to increased LD accumulation and notable changes in droplet area in BV2 cells (Figure 1H, 27.4 ± 5.2 and 36.4 ± 3.8 µm^2^, respectively, n = 5, *p* = 0.013). We also examined the impact of feimin overexpression and knockdown on inflammatory cytokine production (Figure 1I–P). Compared with the untreated control (UC) group, PA treatment significantly increased the expression of inflammatory factors IL‐1β and IL‐6 in cells. Under PA treatment, overexpression of feimin significantly reduced both IL‐1β and IL‐6 mRNA expression (Figure 1I–J, ****p < 0.0001, n = 3 in each group) and their secreted protein levels in the culture medium (Figure 1K,L, *p* = 0.002, 0.009, respectively, n = 5 in each group). Conversely, feimin knockdown led to an increase in IL‐1β and IL‐6 mRNA expression (Figure 1M,N, ****p < 0.0001, n = 3 in each group) and increased levels of secretory proteins of inflammatory factors in the culture medium (Figure 1O,P, *p* = 0.0009, 0.0002, respectively, n = 5 in each group). Together, these findings suggest that feimin may act as a modulator in the PA model by negatively regulating both LD accumulation and the production of inflammatory cytokines, including IL‐1β and IL‐6 in microglia.

### 2.2 Interaction of Feimin and AKT Reduces PA‐Induced LD Accumulation and Inflammation by Suppressing the AKT–mTOR Pathway

**Figure 2:**
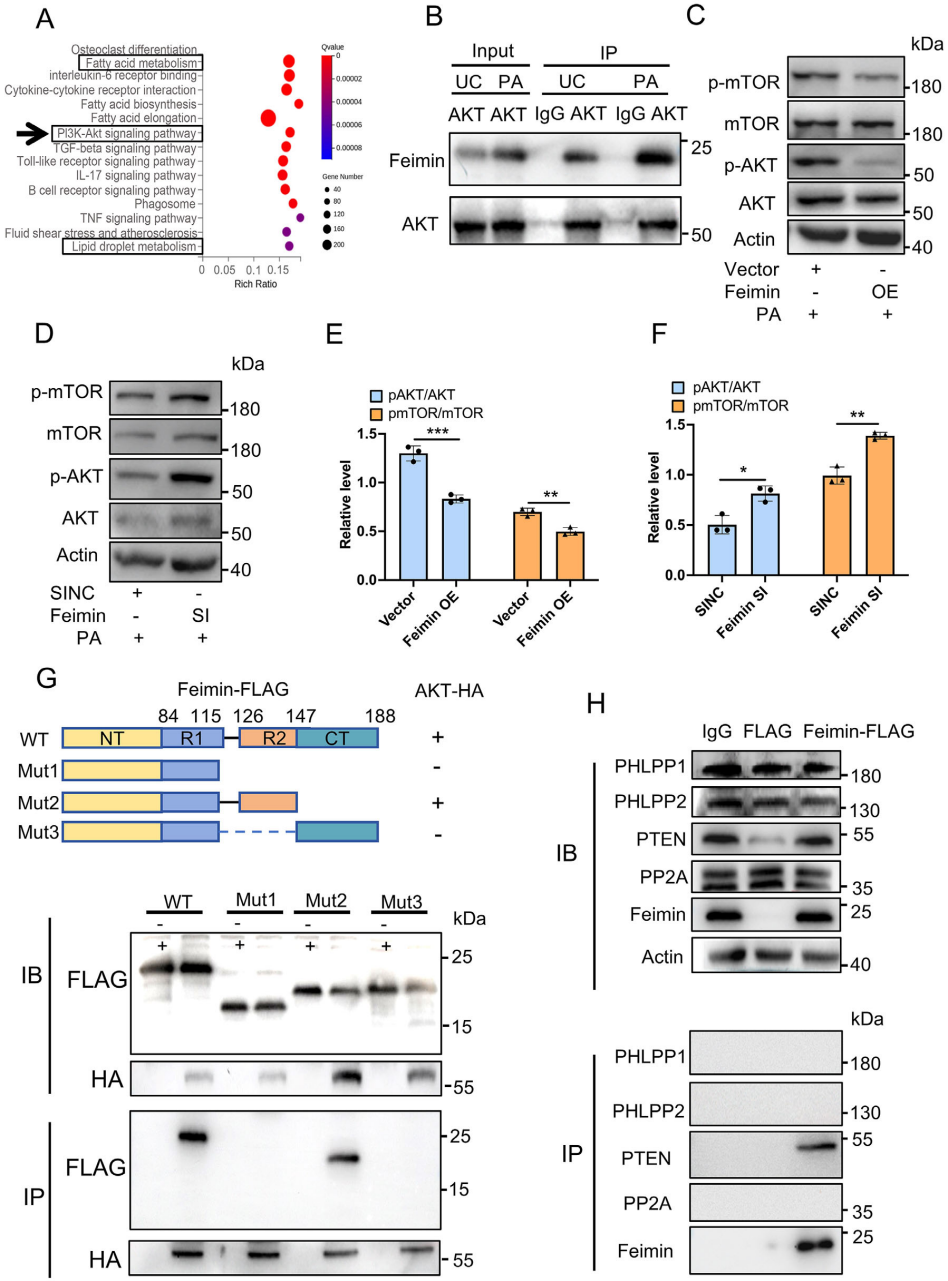
The interaction of feimin and AKT suppresses AKT–mTOR signaling pathway. A) BV2 cells overexpressing feimin, and control cells were stimulated for 24 h with palmitic acid (PA). RNA transcripts were analyzed by RNA sequencing (Vector and feimin OE groups, with n = 3 in each group). The top 15 KEGG enrichment pathways were shown. B) BV2 cells were stimulated for 24 h with PA. Co‐immunoprecipitation (co‐IP) assay was performed with anti‐feimin antibody or control IgG. Immunoblots (IB) in input group (upper panel) and co‐IP group (bottom panel) were determined with anti‐feimin, AKT or GAPDH antibodies, n  =  3 biological replicates. AKT and mTOR proteins and phosphorylation were determined using western blot in BV2 cells with feimin OE C) or feimin SI D) stimulated for 24 h with PA. E,F) GraphPad Prism 9.0 software was used to conduct statistical analysis on the results of Figure C and D, n = 3, *p* = 0.0008 and 0.003, *p* = 0.010 and 0.001. G) Upper: Deletion analysis of the Feimin region required for feimin‐Akt interaction. Bottom: HEK293T cells were co‐transfected with HA‐tagged AKT and different feimin‐FLAG constructs. Cell lysates were immunoprecipitated (IP) using anti‐HA antibodies, followed by immunoblotting (IB) with anti‐FLAG and anti‐HA antibodies. Input blots are shown in the upper panels. Mut: mutant. (H) BV2 cells were transfected with FLAG‐tagged feimin plasmids, and co‐IP assays were performed to assess the interaction between feimin and several classical AKT phosphatases. Cell lysates were immunoprecipitated using anti‐FLAG antibodies, followed by immunoblotting (IB) for PHLPP1, PHLPP2, PTEN, and PP2A. IB: immunoblotting. (B–H) Data were presented as means ± SD of three independent experiments. Two‐tail Student's T‐test.

To further investigate the mechanism by which feimin influences microglial responses in the PA model, we performed a differential expression analysis of feimin OE RNA‐seq data, followed by a Kyoto Encyclopedia of Genes and Genomes (KEGG) enrichment analysis (**Figure** **2**A). The standard QC diagrams (PCA, volcano diagrams) are placed in the Sup Figure 2A,B. We found that the differentially expressed genes were enriched in pathways associated with PI3K‐AKT signaling, fatty acid metabolism, and LD metabolism. The Search Tool for the Retrieval of Interacting Genes (STRING) database analysis identified mTOR as a key interacting protein of AKT (Figure S2C, Supporting Information). According to previous research,^[^ ^29^ ^]^ we know that the AKT‐mTOR pathway plays a key role in both the lipid droplet response and the inflammatory response of microglia. To test the interaction between feimin and AKT, we conducted co‐immunoprecipitation (Co‐IP) experiments in BV2 cells, which showed that under UC conditions, feimin was found to interact with AKT, and this interaction was further strengthened upon PA‐induced lipotoxic stress. (Figure 2B). Notably, feimin overexpression was associated with decreased phosphorylation levels of both AKT and mTOR (Figure 2C,E, *p* = 0.0008, *p* = 0.003, respectively, n = 3 in each group). In contrast, feimin knockdown corresponded to increased phosphorylation of these proteins in the PA model (Figure 2D,F, *p* = 0.010, *p* = 0.001, respectively, n = 3 in each group). Validation of feimin overexpression and knockout in BV2 cells is shown in Figure S2D–G (Supporting Information) (n = 3, *p*  =  0.0003 and 0.042).

To investigate the molecular mechanism by which feimin regulates AKT–mTOR signaling, we explored whether feimin interacts with known AKT phosphatases. Based on previous literature implicating several classical phosphatases such as PP2A, PTEN, PHLPP1, and PHLPP2 in AKT dephosphorylation, we performed both immunoblotting and co‐immunoprecipitation (co‐IP) assays in BV2 microglial cells overexpressing Flag‐tagged feimin. We included three groups: IgG control, Vector‐Flag, and feimin‐Flag. Compared to the Vector‐Flag group, feimin overexpression led to an increase in PTEN levels. Co‐IP assay using an anti‐Flag antibody revealed a specific interaction between feimin and PTEN, a critical lipid phosphatase, to suppress AKT phosphorylation and downstream mTOR signaling. To investigate how feimin regulates AKT expression, we conducted structural mutagenesis and immunoprecipitation experiments to identify the feimin‐binding region. Co‐IP assays confirmed the interaction between feimin and AKT, with a specific region (amino acids 116–146) of feimin being necessary for feimin–AKT association. These findings indicated that feimin suppresses AKT–mTOR signaling by recruiting the lipid phosphatase PTEN and directly interacting with AKT through its 116–146 amino acid region. To assess whether additional signaling pathways are involved in feimin‐mediated modulation of inflammation, we examined NF‐κB and PPARα signaling in BV2 cells. Immunoblot analysis of p‐p65, and PPARα target genes revealed no significant differences between feimin OE and control groups, suggesting that feimin does not regulate these pathways under PA stimulation (Figure S2H,I, Supporting Information).

**Figure 3:**
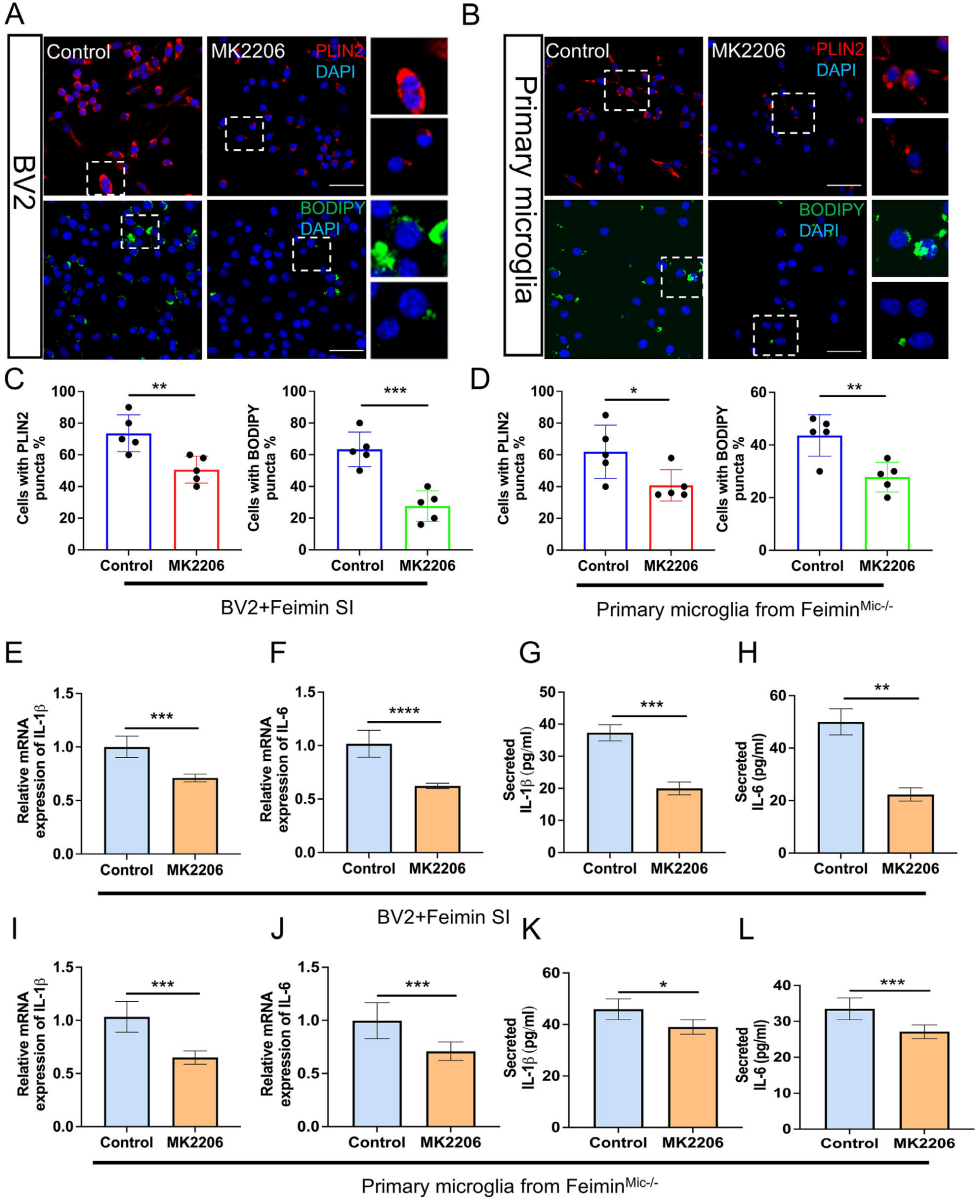
Inhibition of the AKT pathway relieves palmitic acid (PA)‐induced lipid droplet (LD) accumulation and inflammation in feimin‐specific knockdown BV2 cells. A) Feimin‐specific knockdown BV2 cells followed by PA treatment for 24 h and B) The primary microglia from the microglial feimin conditional knockout mice (feimin^Mic‐/−^) after 12 weeks of high‐fat diet (HFD) were pretreated with DMSO or MK2206 (AKT inhibitor, 10 µM) for 30 min, cells were then immunostained for PLIN2^+^/BODIPY^+^(LDs). N = 5, n = 4 biological replicates. Scale bar: 50 µm. C,D) The percentage of cells displaying BODIPY^+^feimin^+^ and PLIN2^+^feimin^+^ puncta was quantified, n = 5, *p* = 0.007 and 0.0006, *p* = 0.041and 0.006, n  =  4 biological replicates. E–H) IL‐1β and IL‐6 mRNA (E, F, *p* = 0.0005, ****p < 0.001) and protein (G, H, *p* = 0.0006 and 0.0032) in feimin siRNA‐treated BV2 cells pretreated with DMSO or MK2206 for 30 min, followed by PA treatment for 24 h, were determined by qPCR and ELISA, respectively. I–L) IL‐1β and IL‐6 mRNA (I, J, *p* = 0.0006 and 0.0004,) and protein (K, L, *p* = 0.033 and 0.0005) in feimin^Mic‐/−^ primary microglia pretreated with DMSO or MK2206 for 30 min, were determined by qPCR (n = 3) and ELISA (n = 5), respectively, n  =  3 biological replicates. (A–L) Data were presented as means ± SD. Two‐tail Student's T‐test.

Previous studies have highlighted the pivotal role of the AKT‐mTOR pathway in regulating LD accumulation and inflammatory responses.^[^ ^29^, ^30^ ^]^ To further explore the involvement of feimin in the PA model through the AKT‐mTOR pathway, we utilized the AKT inhibitor MK2206 or DMSO (as a control) for 30 min.^[^ ^31^, ^32^ ^]^ We assessed its effects in both BV2 and primary microglial cells. We also analyzed the distribution of LDs in BV2 cell lines and primary microglia, using the two LD markers being analyzed: perilipin 2 (PLIN2) and BODIPY dye. In the in vitro PA model, BV2 cells transfected with feimin‐specific siRNA and treated with MK2206 exhibited reduced lipid droplet accumulation (**Figure** **3**A, C, *p* = 0.007 and *p* = 0.0006, respectively, n = 5 in each group). According to the existing literature, LD predominantly accumulates in the cortex and hippocampus.^[^ ^33^ ^]^ For further investigation, we constructed microglia feimin‐conditional knockout mice (feimin^Mic‐/−^) and control mice (feimin^flox/flox^) (Figure S3A,B, Supporting Information) and confirmed the three genotypes (Figure S3C, Supporting Information). In addition, we extracted primary microglia from the hippocampus of adult control and feimin^Mic‐/−^ mice (Figure S3D—F, Supporting Information). We utilized the AKT inhibitor MK2206 or DMSO (Control) for 30 min. Interestingly, MK2206 inhibitor reduced LD accumulation in primary microglia from 12‐week HFD feimin^Mic‐/−^ adult mice (Figure 3B, D, *p* = 0.041, *p* = 0.006, respectively, n = 5 in each group). This reduction in LD accumulation prompted us further to investigate changes in the inflammatory response in PA‐treated microglia. In BV2 cells exposed to PA, the mRNA and culture medium supernatant secretion levels of IL‐1β and IL‐6 were reduced in feimin‐siRNA knockdown cells following MK2206 treatment (Figure 3E–H, *p* = 0.0005, 0.0006, and 0.0032) ****p < 0.0001, n = 3 in each group). A similar trend was observed in primary microglia from 12‐week HFD feimin^Mic‐/−^ adult mice (Figure 3I–L, *p* = 0.0006, 0.0004, 0.033, and 0.0005, n = 3 or 5 in each group). To further enhance generalizability, we extended validation to primary microglia derived from neonatal feimin^Mic‐/−^ mice. Consistent with earlier results, MK2206 treatment resulted in comparable reductions in LD content and IL‐1β and IL‐6 levels relative to control groups (Figure S3G—J, Supporting Information). In summary, our data suggest that feimin may regulate the AKT‐mTOR pathway through its interaction with AKT, thereby influencing LD accumulation and the inflammatory response in microglia within the PA model.

### 2.3 Feimin‐Specific Knockdown in Microglia Triggers Neuronal Apoptosis Through IL‐1β and IL‐6 Secretion in the PA Model

**Figure 4:**
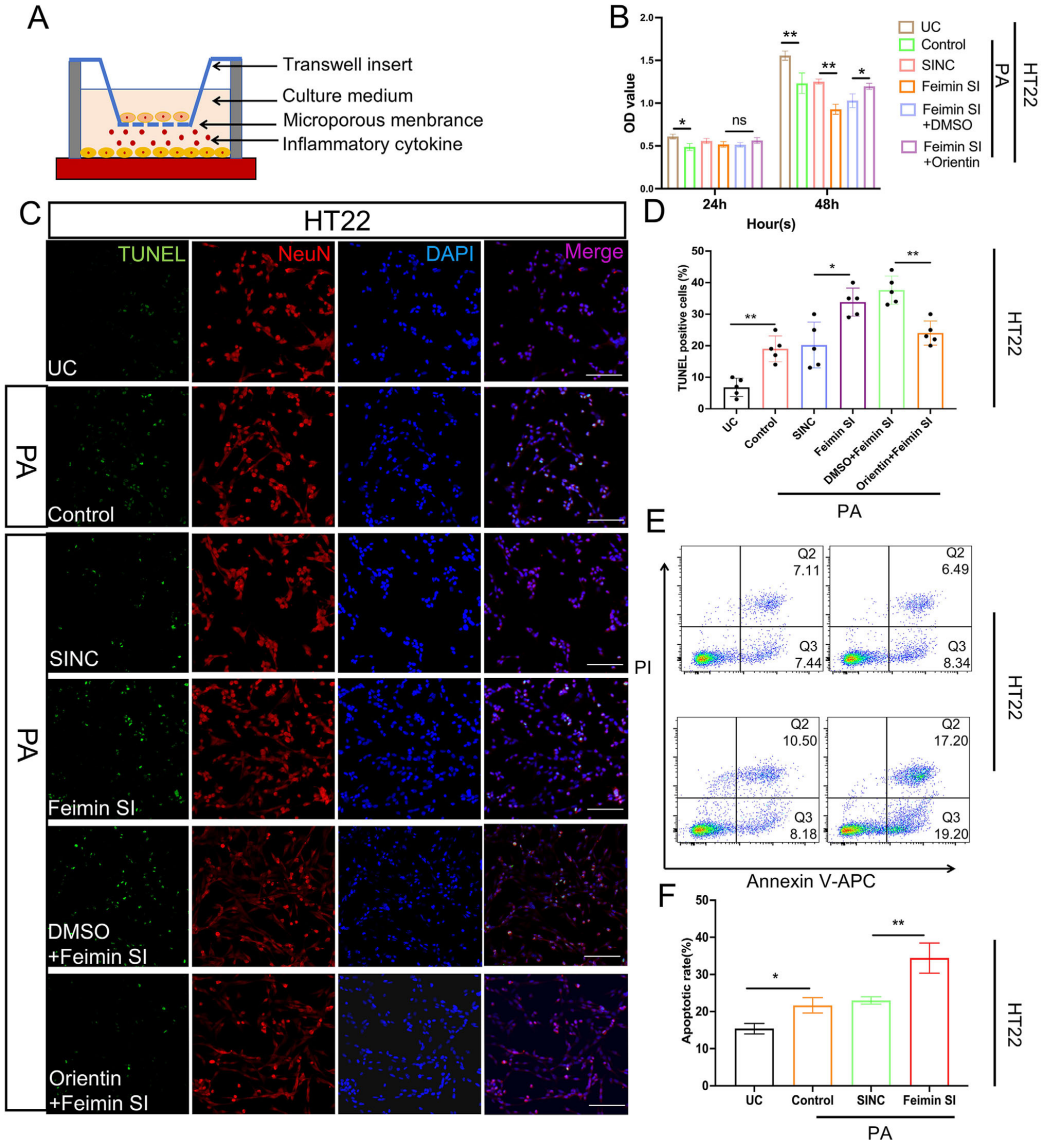
Microglial feimin knockdown promotes neuronal damage by upregulating inflammatory cytokine production. A) Structure diagram of BV2‐HT22 cell interaction model. BV2 cells without or with SINC or feimin SI were pre‐treated without or with palmitic acid (PA) for 24h and then co‐cultured with HT22 cells. B) After 24 and 48 h of co‐culture, the proliferation of HT22 cells was assessed using the CCK8 assay (n = 5). C,D) BV2 cells without or with SINC or feimin SI were pre‐treated without or with PA for 24h and then co‐cultured with HT22 cells with DMSO (control) or orientin (inhibitor for IL‐1β and IL‐6). After 48 h of co‐culture, HT22 cells were stained with TUNEL to assess apoptosis, while NeuN was used as a neuronal marker. Nuclei were counterstained with DAPI (blue). (C) Representative immunofluorescence images and (D) the percentages of TUNEL^+^ HT22 cells were shown. N = 5, *p* = 0.004, 0.011, and 0.001 Scale bar = 50 µm. Control: untreated control group + PA. (E, F) BV2 cells without or with SINC (control) or feimin SI were pre‐treated without or with PA for 24h and then co‐cultured with HT22 cells. After 48 h of co‐culture, apoptosis of HT22 cells was assessed by flow cytometry using Annexin V/7‐AAD double staining (n = 3). E) Representative flow cytometry plots and F) the percentages of Annexin V^+^ apoptotic HT22 cells were shown, *p* = 0.021 and 0.002. (B,D,F) One‐Way ANOVA. (B–F) Data were presented as means ± SD of three independent experiments. *p < 0.05, **p < 0.01, ***p < 0.001.

The activated state of microglia in the PA model triggers the release of pro‐inflammatory and cytotoxic mediators, which promote neuronal apoptosis.^[^ ^34^ ^]^ To investigate the role of feimin in microglia‐mediated neuronal dysfunction, we established a co‐culture model using BV2 cells and HT22 neurons. Initially, BV2 cells were cultured in the upper chamber and treated with feimin‐specific siRNA and PA preconditioning for 24 h, while HT22 cells were seeded in the lower chamber of a 24‐well plate (**Figure** **4**A). PA exposure significantly reduced HT22 cell proliferation compared to the UC group at 48 h, and this reduction was exacerbated when HT22 cells were co‐cultured with feimin‐knockdown BV2 (Figure 4B). TUNEL staining, a technique that labels the ends of DNA fragments, is a widely utilized method for studying neuronal apoptosis.^[^ ^35^ ^]^ TUNEL staining of HT22 cells showed a significant increase in apoptosis in the PA model(control group) compared to the UC group (Figure 4C, D, *p * = 0.004, n = 5 in each group), and this was further amplified in HT22 cells co‐cultured with feimin SI BV2 cells (Figure 4C, D, *p* = 0.011, n = 5 in each group). An APC‐A/7AAD apoptosis assay indicated that HT22 cells co‐cultured with microglial feimin‐specific knockdown had a significantly higher apoptosis rate than that of the control (NCSI) group, both in the early and late stages (Figure 4E, F, *p* = 0.021 and 0.002, respectively, n = 5 in each group). To verify whether feimin mediates neuronal apoptosis through IL‐1β and IL‐6, we added orientin (inhibitor for IL‐1β and IL‐6) or DMSO into the feimin‐knockdown BV2 co‐culture system. Orientin significantly inhibited IL‐1β and IL‐6 production at 40 µm (Figure S5, Supporting Information). In the PA model, orientin 40 µM reduced TUNEL‐positive staining in feimin SI group cells compared to the DMSO solvent group (Figure 4C, D, *p* = 0.001, n = 5 in each group). These findings support the possibility that feimin regulates neuronal apoptosis by modulating the production of IL‐1β and IL‐6.

### 2.4 Microglial Feimin Knockout Aggravates HFD‐Induced Cognitive‐Related Behavioral Disorders Due to the Loss of Suppressive Function in the AKT Pathway

**Figure 5:**
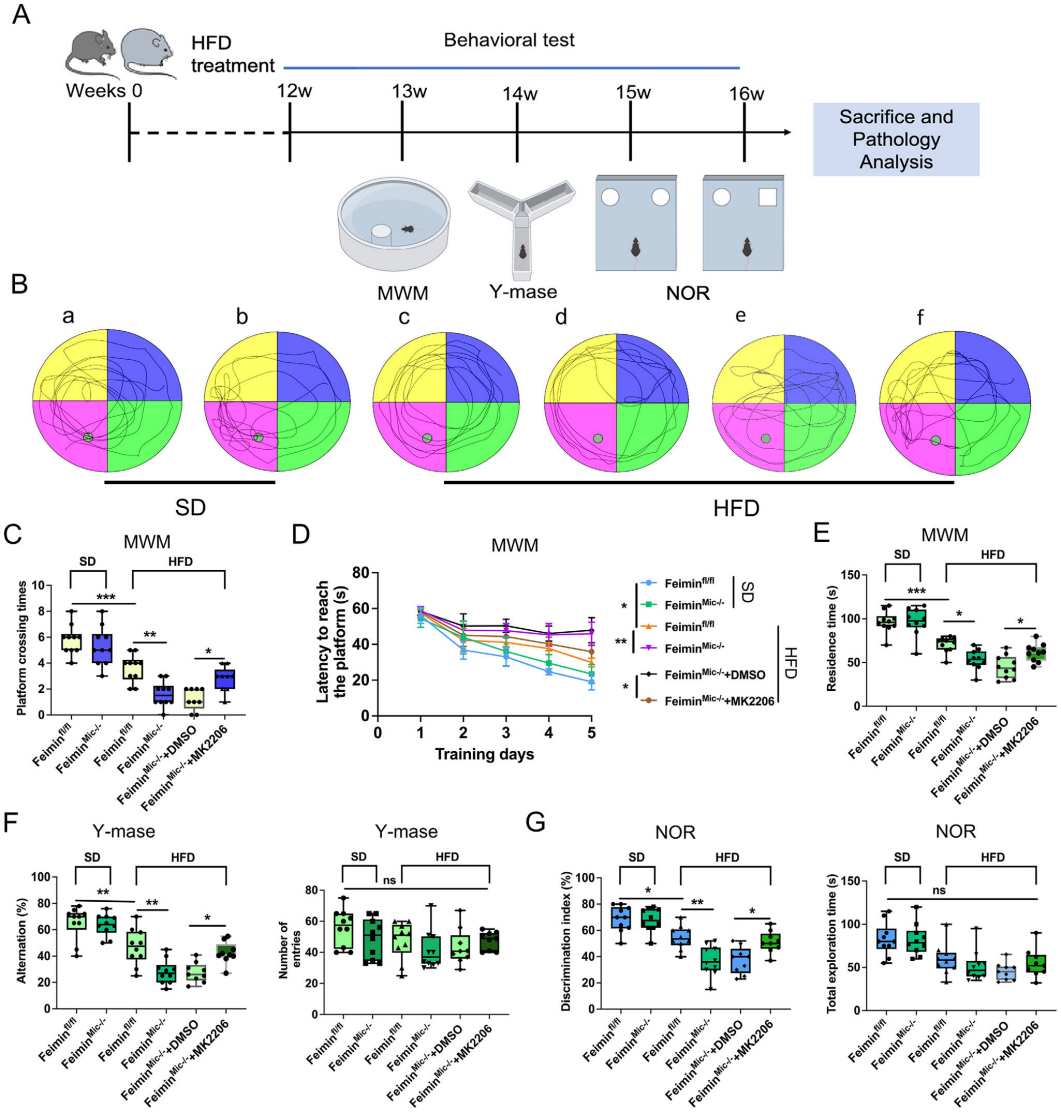
Microglial feimin knockout exacerbates high‐fat diet (HFD)‐induced cognitive behavioral impairment, whereas AKT inhibitor MK2206 reverses the effect of feimin knockout on HFD‐induced cognitive behavioral impairment. A) The experimental design outlines the timeline for animal diet treatment, behavioral testing, and tissue collection. B) Representative positioning and navigation tracks of the Morris Water Maze (MWM) test in the (a) feimin^fl/fl^, (b) feimin^Mic‐/−^, c) HFD + feimin^fl/fl^, d) HFD + feimin^Mic‐/−^, (e) HFD + DMSO + feimin^Mic‐/−^ groups, (f) HFD + MK2206 + feimin^Mic‐/−^ groups. (C) The platform crossing times *p* = 0.0006, 0.002, and 0.042, (D) time to reach the platform during the training period, and E) time spent in the target quadrant in MWM tests were shown *p* = 0.0003, 0.011, and 0.046. Panels C–E show the quantitative analysis of the data in panel B. F) Alternation rate and number of entries in the Y‐maze test *p* = 0.002, 0.002, and 0.038. G) Discrimination index and total exploration time in Novel Object Recognition test *p* = 0.023, 0.003, and 0.037. (B‐G) In each test, n ≥ 9 mice, data were presented as means ± SD of three independent experiments. (C‐G) One‐Way ANOVA, *p < 0.05, **p < 0.01, ***p < 0.001.

It is well established that HFDs significantly impair cognitive function in mice.^[^ ^6^, ^7^ ^]^ To investigate the role of feimin in HFD‐induced cognitive dysfunction, we treated feimin^fl/fl^ (control) and the feimin^Mic‐/−^mice with HFDs for 12 weeks and then administrated a series of behavioral tests (**Figure** **5**A). In the HFD model, wild‐type (WT) and control (feimin^fl/fl^) mice exhibited comparable statistically cognitive performance (Figure S6 (Supporting Information), n = 10, *p* > 0.05). Accordingly, to streamline experimental grouping, feimin^fl/fl^ mice were used as the control cohort in all subsequent studies (Figure S6, Supporting Information). Based on the Morris Water Maze (MWM), Y‐maze, and New Object Recognition (NOR) tests, HFD was associated with impairments in cognitive‐related behaviors (Figure 5B–G, n = 10 in each group).

**Figure 6:**
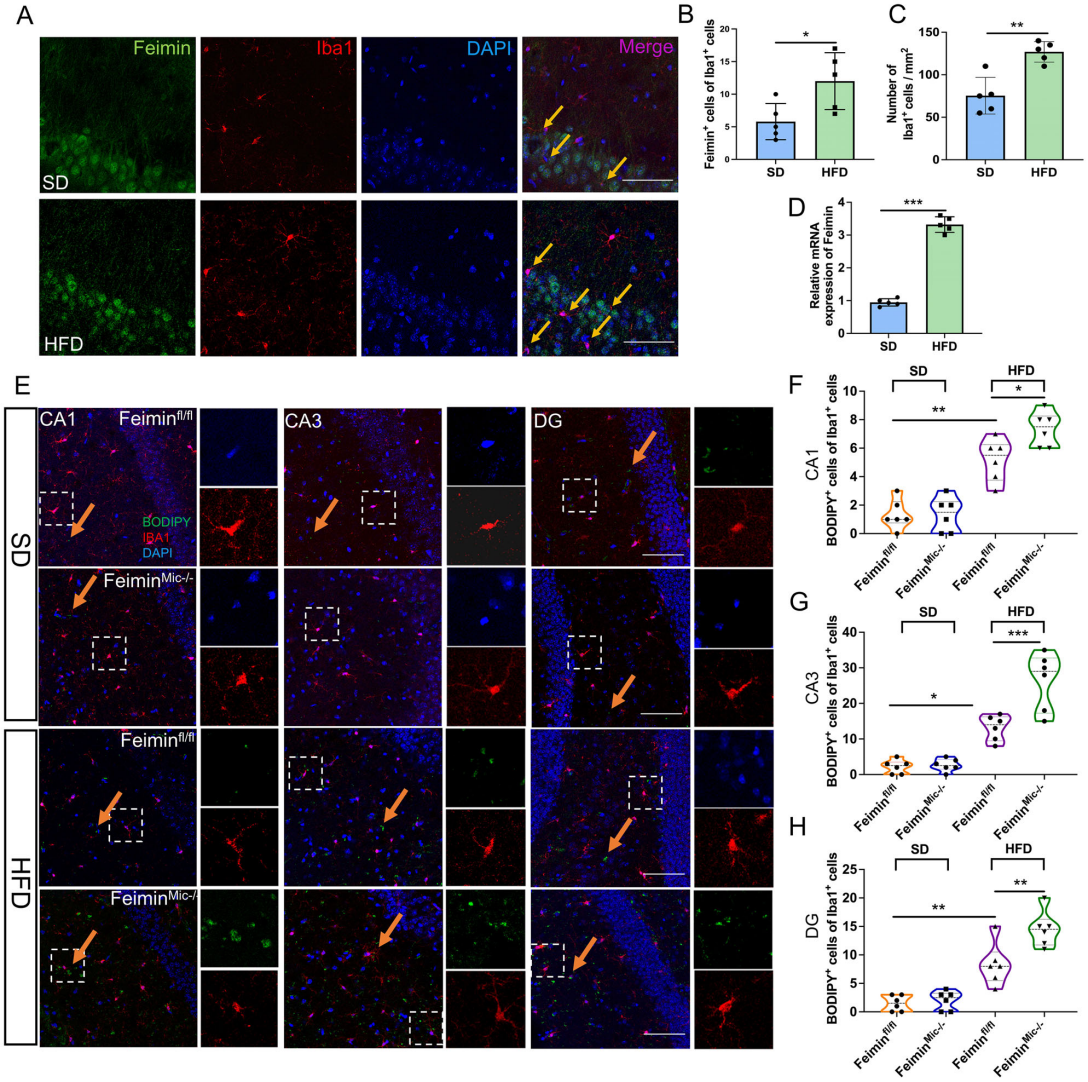
High‐fat diets (HFDs) induce feimin expression, whereas microglial feimin knockout enhances HFD‐induced lipid droplet (LD) accumulation. A) Immunostaining of feimin and IBA1+ (microglia) in the hippocampal CA3 region of control (standard diet [SD]) and HFD mice. Yellow arrow indicates feimin^+^ microglia. n  =  5 mice per group, n  =  3 biological replicates. Scale bar: 50 µm. B) The percentages of feimin^+^ in IBA1^+^ microglia (*p*  = 0.027) and C) the numbers of Iba1^+^ microglia /mm^2^ were shown (*p*  = 0.002). n  =  5 mice per group. D) Feimin mRNA expression was determined by qPCR in the hippocampus of the control and HFD groups (n = 5, *p*  = 0.0003). E) Immunostaining of BODIPY^+^ (LDs) and IBA1^+^ (microglia). Representative LDs are represented by yellow arrows. Scale bar: 50 µm. n  =  6 mice per group. The percentages of BODIPY^+^ in IBA1^+^ microglia from the hippocampi CA1 (F) (n = 6, *p*  = 0.002 and 0.031), CA3 G) (n = 6, *p*  = 0.023 and 0.0004), and DG H) (n = 6, *p*  = 0.004 and 0.003) region from SD+feimin^fl/fl^, SD+feimin^Mic‐/−^, HFD + feimin^fl/fl^, and HFD + feimin^Mic‐/‐^ mice. (A–H) Data were presented as means ± SD of three independent experiments. (B‐D) Two‐tail Student's T‐test, (F–H) One‐Way ANOVA, *p < 0.05, **p < 0.01, ***p < 0.001.

Under standard diet (SD) conditions, the control and the feimin^Mic‐/‐^ mice exhibited no significant difference in all behavioral assays (Figure 5B–G, *p* > 0.05, n = 10 in each group). Following 12 weeks of HFDs, feimin^Mic‐/−^ mice showed impaired spatial memory and recognition, with fewer platform crossings(Figure 5B,C, 5.8 ±1.1 and 3.6 ± 1.1 times, respectively, n = 10, *p*  = 0.0006), longer learning times, reduced target‐quadrant dwell time in the MWM(Figure 5E, 96.20 ±12.42 and 70.80 ± 9.09 s, respectively, n = 10, *p* = 0.0003), lower spontaneous alternation in the Y‐maze (Figure 5F, n = 10, *p* = 0.002), and decreased novel object exploration in the NOR test (Figure 5G, n = 10, *p* = 0.023). These deficits were exacerbated in feimin^Mic‐/−^ mice, which crossed the platform even less frequently(Figure 5B,C, 3.6 ±1.1 and 1.6 ± 1.0 times, respectively, n = 10, *p* = 0.002), exhibited longer learning times, and spent less time in the target quadrant in the MWM(Figure 5D, 70.80 ±9.09 and 53.20 ± 11.30 s, respectively, n = 10, *p* = 0.025), and displayed further reductions in both Y‐maze alternation (Figure 5F, n = 10, *p* = 0.002) and NOR exploration (Figure 5G, respectively, n = 10, *p*  =  0.003), without affecting the locomotor activity(Figure 5G, I, *p* > 0.05). Notably, there was no significant difference in food intake between the control and the feimin^Mic‐/−^ mice under SD and HFD conditions (Figure 6, n = 10, *p* > 0.05). To investigate the contribution of AKT signaling, feimin^Mic‐/−^ mice were treated with either vehicle DMSO or the AKT inhibitor MK‐2206. MK‐2206 partially restored cognitive‐related behavioral disorders in HFD feimin^Mic‐/−^ mice, increasing platform crossings (Figure 5B,C 1.2 ±0.8 and 2.8 ± 1.0 times, respectively, n = 9, *p* = 0.042)and shorter learning times, and increased target‐quadrant dwell time in the MWM (Figure 5E 45.00 ± 13.02 and 61.30 ± 10.20 s, respectively, n = 10, *p* = 0.046), enhanced Y‐maze alternation (Figure 5F, n = 9, *p* = 0.038), and improving NOR discrimination and total exploration time (Figure 5G, n = 9, *p* = 0.037).

Collectively, compared with SD mice, HFD mice exhibited marked impairments in cognitively determined behaviors. Microglia‐specific knockout of feimin further exacerbated the initial behavioral deficits, whereas treatment with an AKT inhibitor significantly alleviated HFD‐induced symptoms of cognitive behavioral disorders in feimin^Mic‐/−^ mice.

### 2.5 Microglial Feimin Knockout Aggravates HFD‐Induced LD Accumulation in the Hippocampus

To investigate the impact of feimin expression in microglia, we first examined the feimin distribution in brain microglia of WT mice and found that feimin was expressed in the medulla oblongata, cortex, striatum, and hypothalamus ― most frequently in the hippocampus (Figure S7A, Supporting Information). As HFD can affect multiple brain regions, including the hippocampus, prefrontal cortex, and hypothalamus,^[^ ^33^ ^]^ we examined the distribution of feimin in these areas of WT mice using immunofluorescence. We observed the obvious expression of feimin^+^ microglia in these regions, especially in the CA3 region (Figure S7B, Supporting Information). We performed confocal microscopy to examine the hippocampal CA3 region of mice fed HFDs for 12 weeks (**Figure** **6**A). HFD induced a significant increase in microglial feimin expression and Iba1^+^microglia cells (Figure 6B,C, n = 5, *p*  = 0.027 and 0.002, respectively). We also confirmed this through the mRNA expression in the hippocampus tissue of HFD mice after 12 weeks (Figure 6D, n = 5, *p*  = 0.0003). It has been reported that LDs are primarily distributed throughout the brain, mainly on microglia in the cortex and hippocampus, but not in astrocytes or neurons.^[^ ^32^ ^]^ Under the SD model, LD accumulation in the hippocampus was relatively small, with no significant difference between control and feimin^Mic‐/−^ mice (Figure 6E–H, n = 6, *p* > 0.05). Moreover, compared to the feimin^fl/fl^ group, feimin^Mic‐/−^ mice demonstrated higher levels of BODIPY^+^LD and Iba1^+^microglia in the hippocampal CA1, CA3, and DG, especially in the CA3 region (Figure 6E–H, n = 6, *p*  = 0.031,0.0004 and 0.003, respectively). This was further confirmed using PLIN2^+^ LD (Figure S8C, D, n = 5, *p*  = 0.011,0.0003 and 0.004, respectively, Supporting Information). In addition, after 12 weeks of HFD, we assessed LD accumulation in the hypothalamus and prefrontal cortex of HFD mice and found no differences between control and feimin^Mic−/−^ mice (Figure S8E, F, n = 5, *p* > 0.05, Supporting Information). Therefore, we focused our investigation on the hippocampus, a region closely associated with cognition. These results suggest that microglial feimin plays a vital role in the HFD model, and microglial feimin knockout intensifies HFD‐induced LD accumulation in the hippocampus.

### 2.6 Microglial Feimin Conditional Knockout Promotes HFD‐Induced Neuronal Damage by Enhancing IL‐1β and IL‐6 Production

**Figure 7:**
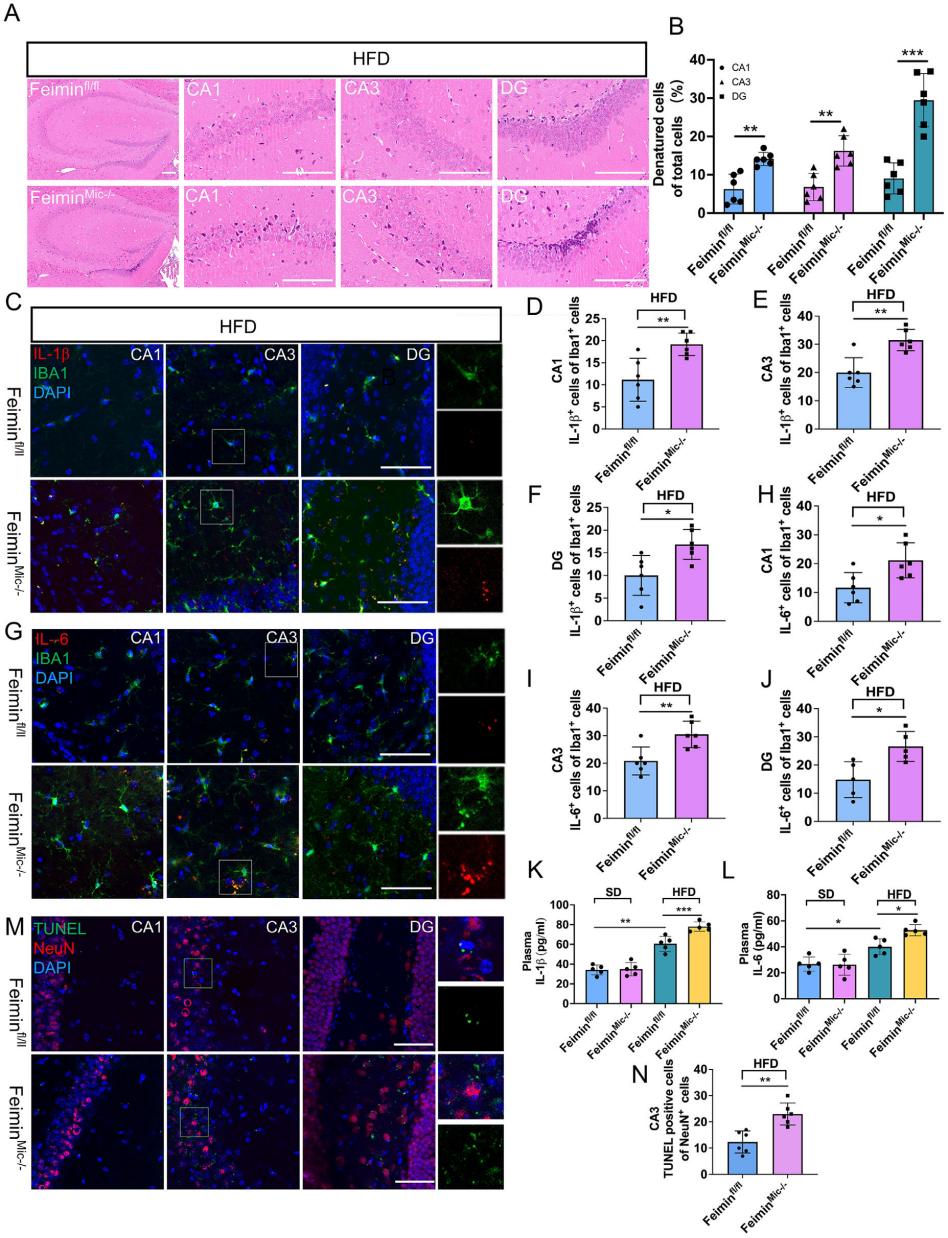
Microglial feimin knockout promotes high‐fat diet (HFD)‐induced inflammatory factor accumulation and neuronal damage in the hippocampus. A,B) Representative hematoxylin‐eosin (HE) staining of the hippocampus and quantification of degenerated cells as a percentage of total cells in the DG region of feimin^fl/fl^ and feimin^Mic–/–^ mice after 12 weeks of HFD treatment (n = 6, *p*  = 0.002, 0.004, 0.0004, Scale bar: 50 µm). C–J) Immunofluorescence staining of IL‐1β (C) or IL‐6 (G) and IBA1 in the CA1, CA3, and DG regions of HFD‐treated feimin^fl/fl^ and feimin^Mic‐/−^mice. scale bar: 50 µm. The percentages of IL‐1β^+^ (D‐F, n = 6, *p*  = 0.005,0.001and 0.012) and IL‐6^+^ (H‐J, n = 6, *p* = 0.015,0.006, and 0.013) in IBA1^+^ cells in the CA1 (D, H), CA3 (E, I), and DG (F, J) regions of HFD‐treated feimin^fl/fl^ and feimin^Mic‐/−^mice. K,L) Plasma (K, n = 5, *p*  = 0.002 and 0.0002) IL‐1βand (L, n = 5, *p*  = 0.017 and 0.021) IL‐6 protein concentrations were determined by ELISA. M) Immunofluorescence staining of TUNEL and NeuN from CA1, CA3, DG regions of HFD‐treated feimin^fl/fl^ and feimin^Mic‐/−^mice. NeuN was used as a neuronal marker. Nuclei were counterstained with DAPI (blue), n = 6, Scale bar: 50 µm. N) The percentages of TUNEL^+^ in NeuN^+^ cells from CA3 of HFD‐treated feimin^fl/fl^ and feimin^Mic‐/−^mice (n = 6, *p*  = 0.001). (A‐M) Data were presented as means ± SD of three independent experiments. (D‐J, N) Two‐tail Student's T‐test, (K, L) One‐Way ANOVA, *p < 0.05, **p < 0.01, ***p < 0.001.

Under SD conditions, hematoxylin and eosin staining (HE) staining of hippocampal sections (CA1, CA3, DG) indicated normal neuronal number and morphology, with no significant differences observed between feimin^fl/fl^ and feimin^Mic‐/−^ mice (Figure S9A, n = 5, *p* > 0.05, Supporting Information). While under HFD conditions, feimin^fl/fl^ mice exhibited mild cellular irregularities and nuclear condensation. These abnormalities were more pronounced in feimin^Mic‐/−^ mice, which exhibited substantial neuronal loss, cell shrinkage, and severe cytoplasmic and nuclear edema, with marked abnormalities in nuclear membrane integrity and intensified nuclear staining. Especially in the DG region, the proportion of degenerated cells in the tissues of feimin^Mic‐/‐^ mice increased (**Figure** **7**A, B n = 6, *p* = 0.0004). Immunofluorescent analysis was performed further to assess the effect of feimin conditional knockout on neuroinflammation. This analysis revealed no statistical difference in inflammatory factors between SD groups of control and feimin^Mic‐/−^ mice (Figure S9B,C, n = 6, *p* > 0.05, Supporting Information). After 12 weeks of an HFD, feimin^Mic‐/−^ mice showed a significant increase in the percentage of IL‐1β^+^ and IL‐6^+^ cells in Iba1^+^microglia in the hippocampal CA1, CA3, and DG regions compared with feimin^fl/fl^ controls. (Figure 7C–J, n = 6). Since the number of microglia correlates with their function, we further analyzed the microglial population in the CA3 region. We observed significantly higher numbers of IL‐1β^+^IBA1^+^ and IL‐6^+^IBA1^+^ cells in feimin^Mic‐/−^ mice than that in feimin^fl/fl^ mice (Figure S9D,E, n = 6, *p*  = 0.03 and 0.001, respectively, Supporting Information). To examine possible differences in inflammatory cytokines due to HFDs in feimin^fl/fl^ and feimin^Mic‐/−^ mice, plasma samples were also assessed via ELISA. Plasma concentrations of IL‐1β and IL‐6 were elevated in HFD mice compared to SD mice (Figure 7K,L, n = 5, *p*  = 0.002 and 0.017, respectively), with even higher levels observed in HFD feimin^Mic‐/−^ mice than HFD feimin^fl/fl^ mice (Figure 7K,L, n = 5, *p*  = 0.0002 and 0.021, respectively). Finally, we explored the impact of an HFD on hippocampal neuronal apoptosis, which is associated with cognitive decline. In the HFD model, feimin^Mic‐/−^ mice showed a significant increase in TUNEL^+^ neurons in the hippocampal CA1, CA3, and DG regions compared to the control mice (Figure 7M,N, n = 6, *p*  = 0.001). Taken together, like the in vitro experiments, these results indicate that microglial feimin knockout exacerbates HFD‐induced neuronal damage, possibly through the increased production of IL‐1β and IL‐6.

### 2.7 A longitudinal Study on the Expression and Function of Feimin

To extend our findings from the 12‐week HFD experiments into a longitudinal framework, we selected an early time point after HFD initiation—three days, based on previous literature—to evaluate the effects of short‐term HFD on feimin expression and function.^[^ ^36^, ^37^ ^]^ Our results showed that at HFD day 3, feimin expression in control mice (feimin^fl/fl^) was not significantly different from that in SD control mice (Figure S8A, n = 5, Supporting Information). In contrast, feimin expression in control mice were significantly increased after 12 weeks of HFD compared with data from HFD day 3 (Figure S8A, n = 5, Supporting Information). At the early stage of HFD, neither control nor feimin^Mic–/–^ mice exhibited appreciable LD accumulation (Figure S8B, n = 5, Supporting Information). Although the pro‐inflammatory cytokines IL‐1β and IL‐6 were expressed, their levels did not differ significantly (Figure S9F, G, n = 6, Supporting Information).

Collectively, these findings suggest that feimin may not play a prominent role during the early stages of HFD exposure. As the HFD duration increases, progressive LD accumulation triggers a significant rise in pro‐inflammatory cytokines, which in turn is accompanied by robust induction of feimin expression.

## 3 Discussion

**Figure 8:**
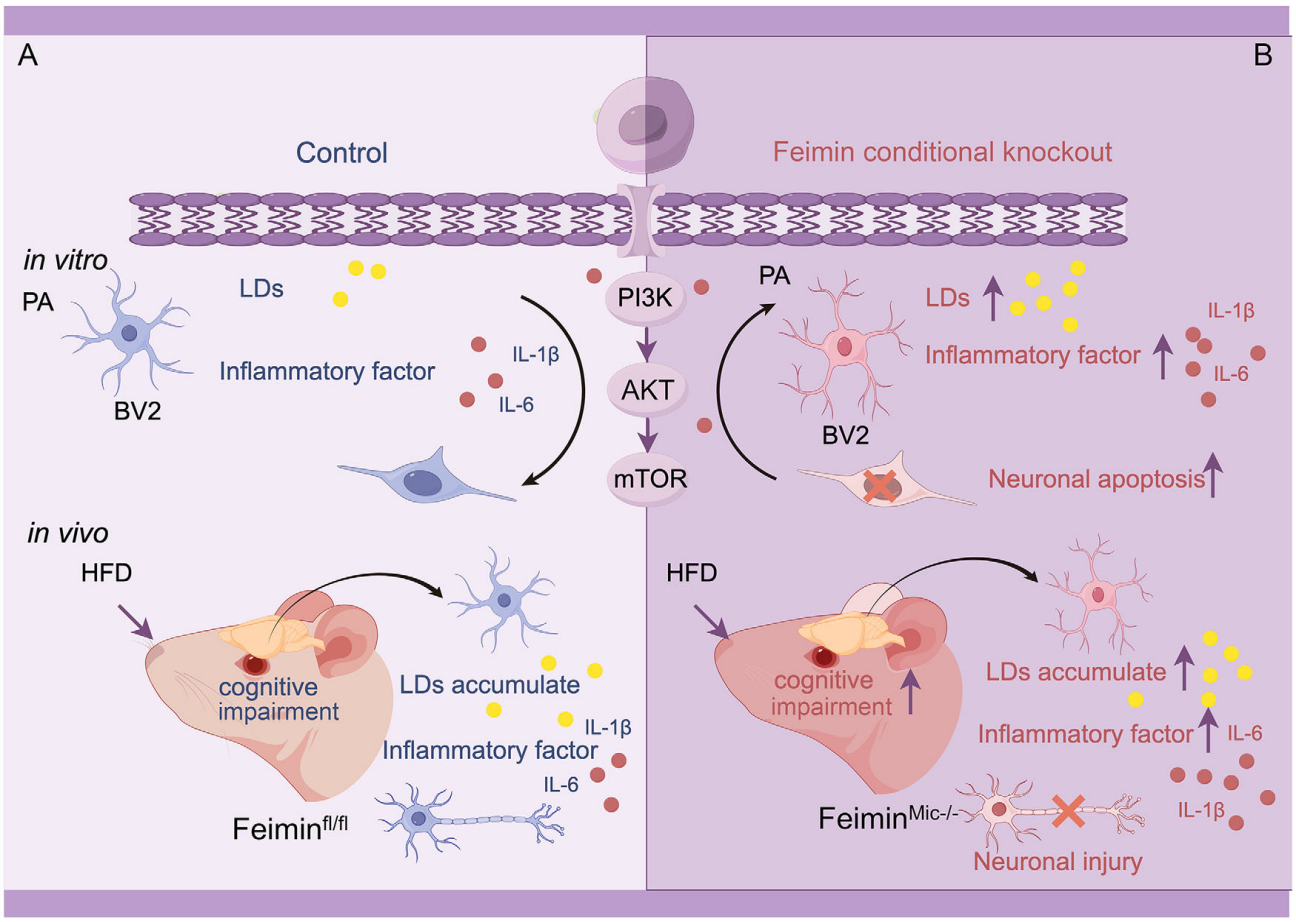
Schematic of the mechanism by which microglial feimin deletion triggers microglial reactivity in the lipotoxicity model. A) In the control group of the in vitro model, PA stimulation led to increased lipid droplet (LD) accumulation in BV2 cells, triggering the upregulation of inflammatory factors IL‐1β and IL‐6, which in turn induced neuronal damage. Similarly, in the feimin^fl/fl^ group of the in vivo model, high‐fat diet (HFD) exposure resulted in LD accumulation in the hippocampus, elevated IL‐1β and IL‐6 levels, increased neuronal apoptosis, and impaired cognitive function in mice. B) In both in vitro and in vivo models with microglial feimin knockout under HFD conditions, these effects were further exacerbated and acted through the PI3K‐AKT‐mTOR pathway, ultimately leading to severe cognitive impairment in mice.

Clinically, HFDs are frequently associated with metabolic disorders. A serious complication in patients with metabolic disorders is cognitive impairment—the decline or loss of attention, memory, perception, and executive ability, leading to dysfunction and reduced quality of life.^[^ ^6^, ^38^ ^]^ Lipid accumulation and chronic neuroinflammation causes play a critical role in the onset and progression of cognitive disorders.^[^ ^16^, ^39^ ^]^ In this study, we investigated the role of feimin in microglia and provided evidence supporting its regulatory role in LD accumulation and neuroinflammatory responses under lipotoxic stress. Our findings suggest that the absence of feimin exacerbates LD accumulation and inflammatory damage in microglia and neuronal damage, both in vitro and in vivo. Furthermore, the loss of feimin expression in microglia significantly worsens HFD‐induced cognitive behavioral impairment. These insights have important implications for understanding the underlying mechanisms of HFD‐related cognitive dysfunction (**Figure** **8**).

HFDs are known to elevate circulating levels of free fatty acids (FFAs), including saturated palmitates, which have been shown to affect the peripheral immune system.^[^ ^40^ ^]^ LDs are widely associated with inflammation, as they act as inflammatory organelles in peripheral immune cells, such as foam macrophages in atherosclerosi.^[^ ^41^ ^]^ As the primary immune cells of the CNS, microglia, in recent years, have garnered growing attention in this context. Studies have shown that conditions such as Alzheimer's disease (AD) and aging lead to significant “LD accumulation in microglia,”, a phenomenon referred to as LD accumulation in microglia (LDAM).^[^ ^17^, ^42^ ^]^ Previous studies have shown that under aging or metabolic stress, LD accumulation in the CNS occurs predominantly in microglia.^[^ ^17^, ^33^ ^]^ For instance, Li et al. reported that in db/db mice, LDs were almost exclusively localized to microglia, with negligible presence in astrocytes and neurons.^[^ ^33^ ^]^ Consistent with these findings, our work has primarily focused on microglia. In recent years, several key molecules have been identified as critical regulators of microglial lipid metabolism and inflammatory responses, including TREM2, APOE, and CD36.^[^ ^43^, ^44^ ^]^ Unlike TREM2 and APOE, which mainly affect lipid uptake or transport, feimin is a lipotoxic stress–induced regulator that links lipid metabolism to inflammatory signaling via AKT, acting as a downstream integrator of metabolic stress and microglial activation.

Shi *et al.* identified that feimin is a key regulator of glucose homeostasis and exercise performance.^[^ ^23^ ^]^ Feimin modulates glucose metabolism by promoting glucose uptake and inhibiting glucose production in target tissues, thus influencing muscular thermogenesis during exercise.^[^ ^23^ ^]^ While that study emphasized the role of feimin in peripheral metabolic regulation, metabolic alterations are also a hallmark of HFD exposure. Our team was the first to discover significant downregulation of feimin in microglia during stroke and found that feimin plays a role in reducing inflammatory factors in stroke microglia.^[^ ^25^ ^]^ These studies suggest that feimin plays an important role in both peripheral and central areas. Our temporal analyses indicate that feimin does not influence the early hippocampal response to HFD‐induced lipotoxicity. Instead, feimin expression progressively increases and plays a more prominent role during the chronic phase of lipid overload, as evidenced by the marked differences observed after 12 weeks of HFD feeding. These findings suggest that feimin may act as a late‐stage regulator, activated as a reactive response to LD accumulation, thereby contributing to the persistence or amplification of metabolic dysfunction and inflammation. From a therapeutic perspective, targeting feimin may be more effective after metabolic disturbances have developed.

To investigate feimin's effect on microglia in a PA model, we found that overexpression of feimin increased LD accumulation, while feimin knockdown was associated with reduced LD formation. LD accumulation under PA conditions is closely linked to inflammation,^[^ ^17^ ^]^ prompting us to focus on the inflammatory response. Our results showed that IL‐1β and IL‐6 levels were significantly reduced in feimin‐overexpressing microglia, while the opposite was true in feimin‐knockdown microglia. Feimin protects against LD accumulation and inflammation. Its increase in the PA model may be a response to heightened LD levels and inflammation, aiding in damage clearance.

Regarding the role of feminin in clearing LDs and reducing inflammation, we found through differential expression analysis of RNA‐seq data that the PI3K‐AKT pathway may play an important role. Pi *et al.* reported that P2RY12 receptor activation promotes the formation and lipid accumulation of VSMC‐derived foam cells through the PI3K‐AKT‐mTOR signaling pathway.^[^ ^30^ ^]^ mTOR is a major downstream effector of the PI3K/AKT pathway.^[^ ^45^ ^]^ In the PA model, feimin overexpression led to a significant reduction in phosphorylated AKT and mTOR levels compared to the control group, while feimin depletion had the opposite effect. Our findings suggest that feimin mediates the PA model through the AKT–mTOR pathway. Under PA conditions, feimin and AKT binding was further strengthened. The binding kinetics of this interaction remain to be elucidated; meanwhile, our results provide new insights into how feimin interacts with AKT. We identified a discrete binding domain within feimin (amino acids 116–146) that is essential for its strong association with AKT. We further observed that feimin recruits the AKT phosphatase PTEN, suggesting that it may function as a scaffold to promote AKT dephosphorylation and attenuate downstream signaling. Compared with other phosphatases, PTEN is a canonical lipid phosphatase that specifically dephosphorylates PIP^3^ to suppress AKT activation, and it plays a central role in regulating microglial lipid metabolism and inflammatory responses.^[^ ^46^ ^]^ To further determine the specific role of the AKT pathway, we added the AKT inhibitor MK2206 to feimin‐knockdown microglia. We were surprised to find that LD accumulation and inflammatory cytokines (IL‐1β and IL‐6) were significantly reduced in both BV2 cells and primary microglia. These results provide preliminary evidence of feimin's role in the PA response in vitro. In the PA model, feimin overexpression and knockdown in BV2 cells decreased and increased AKT and mTOR phosphorylation, respectively, thereby inhibiting or promoting LD accumulation and inflammation, respectively.

It has been reported that high‐fat models are often accompanied by neuronal apoptosis, and alterations in neuron‐microglia communication can lead to brain disorders, affecting synaptic function and neuronal survival.^[^ ^47^, ^48^ ^]^ Microglia influence neurons through inflammatory responses and neurotrophic factors.^[^ ^49^ ^]^ In the PA model, co‐culturing feimin‐specific microglia with neurons was associated with increased neuronal damage. We hypothesized that feimin‐specific microglia cause damage through the secretion of inflammatory cytokines IL‐1β and IL‐6. Adding orientin (the IL‐1β and IL‐6 inhibitor) to feimin‐specific knockdown microglia significantly reduced the number of TUNEL⁺ HT22 (NeuN⁺) neurons, further affirming our hypothesis that excessive secretion of IL‐1β and IL‐6 from feimin‐deficient microglia may contribute to neuronal injury under lipotoxic conditions.

Neuronal apoptosis is often associated with mental disorders and cognitive impairments in both human and animal models.^[^ ^50^ ^]^ HFD feimin^Mic‐/−^ mice exhibited more severe cognitive dysfunction and significant neuronal apoptosis. LD accumulation in the hippocampus microglia of T2DM mice and HFD mice with cognitive dysfunction has long been reported.^[^ ^22^ ^]^ In our research, we specifically observed more significant LD accumulation, increased IL‐1β and IL‐6 levels, and a rise in microglial numbers in these mice. These results align with our in vitro data. In the HFD feimin^Mic‐/−^ mice, microglial LD accumulation led to increased inflammatory factors, resulting in significant neuronal apoptosis and severe cognitive dysfunction. However, treatment with AKT inhibitors in HFD feimin^Mic‐/−^ mice improved cognitive function. Studies have shown that abnormal PI3K/AKT pathway activation in AD correlates with cognitive decline.^[^ ^51^ ^]^ This further supports the importance of AKT's role in feimin‐ triggered cognitive behaviors.

Recent evidence indicates that the detrimental effects of HFD on behavior and hippocampal neuroplasticity are largely driven by shifts in microglial phenotype, most notably a pronounced accumulation of LDs within these cells.^[^ ^16^, ^33^ ^]^ In this study, we demonstrated that feimin may act as a key regulator of LD homeostasis, inflammatory signaling, and the consequent cognitive and behavioral deficits mediated by microglia.

The human ortholog of feimin, C5orf24, has been notably implicated across a range of neuropsychiatric and inflammatory conditions, underscoring its translational potential. Whole‐genome expression analysis has identified feimin as one of the downregulated genes associated with symptom improvement in PTSD, of which hippocampal cognitive impairment is a prominent clinical feature.^[^ ^52^, ^53^ ^]^ A separate GWAS (genome‐wide association study) comparing 527 Parkinson's disease patients and 435 healthy controls linked feimin variants specifically to early‐onset PD (<50 years of age).^[^ ^54^ ^]^ Mendelian randomization further suggests that genetically elevated feimin levels confer protection against ischemic stroke.^[^ ^25^ ^]^ While our data support a role for feimin in the regulation of neuroinflammation and neurodegeneration in murine models, its translational potential remains to be established. Rigorous validation in human systems, such as iPSC‐derived microglia and postmortem brain tissue from relevant patient cohorts, will be essential to confirm its dysregulation and function. Building on this foundation, future studies should evaluate the efficacy, safety, and clinical feasibility of feimin‐targeted interventions, including small‐molecule modulators, RNA‐based therapeutics, and microglia‐specific delivery platforms, to enhance feimin function in disease‐relevant contexts. However, some limitations remain. First, although we identified a tight interaction between the feimin domain (amino acids 116–146) and AKT, the binding kinetics remain to be further elucidated. Second, the specific mechanisms of neuronal damage require further investigation. Third, the use of Cx3cr1‐Cre mice to achieve microglia‐specific deletion of feimin may introduce off‐target effects that cannot be completely excluded. Finally, future investigations should evaluate the relevance of feimin in human disease and assess its feasibility for clinical translation.

## 4 Conclusion 

Our findings uncover the mechanism of feimin in microglia in the hyperlipid‐induced model, showing that feimin knockdown leads to neuronal damage and cognitive impairment by regulating LD accumulation and inflammation in hippocampal microglia, in which the AKT‐mTOR pathway is involved. Thus, microglial feimin may be a key therapeutic target for HFD‐induced cognitive impairment.

## 5 Experimental Section

### Mice

Cx3cr1‐Cre and feimin^flox/flox^(feimin^fl/fl^) mice were purchased from Cyagen. They generated feimin^fl/fl^ mice by inserting loxP sites flanking Exon 2 of the feimin gene. (Figure S3A, Supporting Information). Dot plot self‐alignment revealed no significant tandem repeats. GC content analysis showed no regions of high GC density. Both results indicate that this region is suitable for PCR screening and sequencing. A BLAT search of the 3 kb regions upstream and downstream of Exon 2 identified no significant off‐target similarity in the genome (Figure S3B, Supporting Information). We backcrossed to the C57BL/6J background for more than six generations. Microglia feimin‐conditional knockout mice (hereafter referred to as feimin^Mic‐/−^) were produced by mating feimin^fl/fl^ mice with Cx3cr1‐Cre mice. We performed genotyping by PCR (Figures S3B and 4, Supporting Information). All mice were housed in a specific pathogen‐free (SPF) animal facility at Capital Medical University, where environmental parameters (temperature, humidity, light/dark cycle) and microbial status are strictly regulated. Veterinary staff regularly monitored the animals to ensure their well‐being and to identify any signs of disease or distress. As described in previous studies,^[^ ^55^, ^56^ ^]^ mice were fed either a standard diet (SD) or a high‐fat diet (HFD) (Research Diet, D12492, USA) for 12 weeks. Additionally, all animals had free access to food and water throughout the experiment unless otherwise specified. All animal procedures adhered to the guidelines of the Animal Ethics Committee of Capital Medical University, and the experimental protocols were approved accordingly.

### RNA Extraction and qPCR Assay

Total RNA was extracted from purified microglia using the BV2 Cell/Primary Microglia/Tissue Total RNA Isolation Kit V2 (Vazyme, RC112‐01). The extracted RNA was then reverse‐transcribed into complementary DNA (cDNA) using the HiScript III 1st Strand cDNA Synthesis Kit (Vazyme, R323‐01). qPCR was performed on a Roche LightCycler 480 PCR System using Hieff UNICON qPCR SYBR Green Mastermix (Shanghai YEASEN Biotech, 11198ES). Gene expression levels were analyzed using the 2−ΔΔCt method and normalized to β‐actin. The qPCR primers are listed in Sup Table **1**.

### Cell Culture

BV2 murine microglial cells (Procell, CL‐0493A) and HT22 were cultured in Dulbecco's modified Eagle's medium (DMEM) supplemented with 10% FBS and 1% PS. As described in the previous study, the medium was switched to DMEM containing 200mM PA (final working concentration) for treatment.^[^ ^38^, ^57^ ^]^ A fatty acid‐albumin complex solution containing PA (PA, Sigma‐Aldrich, St. Louis, MO, USA) and fatty acid‐free bovine serum albumin (BSA, Sigma, St. Louis, MO, USA) was prepared as previously described.^[^ ^58^ ^]^

To achieve stable overexpression of feimin in BV2 cells, BV2 cells were seeded in culture plates one day prior to transduction. We used the lentivirus constructed and packaged by VectorBuilder Company (Guangzhou, China) Company for lentiviral transduction. Details of the lentiviral construct are provided in the Supplementary Figures. Transduction was performed at a multiplicity of infection (MOI) of 50. After 6 h of incubation with the virus‐containing medium, the medium was replaced with fresh complete medium. Forty‐eight h post‐transduction, GFP fluorescence intensity was monitored using fluorescence microscopy. Transduction efficiency was assessed by comparing the percentage of EGFP‐positive cells and FACS relative to untransduced BV2 cells, achieving ≈90–96% efficiency. Following transduction, cells were selected with puromycin (2 µg ml^−1^) to establish stable feimin‐overexpressing cell lines. Puromycin was withdrawn one day prior to subsequent experiments or RNA sequencing, and the cells were reseeded for further assays. RNA sequencing was performed on the BGISEQ‐500 platform (BGI‐Shenzhen, China) to generate paired‐end reads. Differentially expressed genes (DEGs) were identified using DESeq2 with the thresholds of |log_2_ (fold change) | ≥ 1 and adjusted *p*‐value (Benjamini–Hochberg FDR) < 0.05. KEGG pathway enrichment analysis of DEGs was performed using the clusterProfiler R package, with a significance threshold of adjusted *p*‐value < 0.05. Pathways meeting these criteria were considered significantly enriched. In BV2 cells, siRNA‐mediated knockdown of feimin was performed in BV2 cells using Lipofectamine RNAiMAX (Invitrogen, 13778150), following the manufacturer's protocol. Feimin‐targeting siRNAs were synthesized by Ribobio Biosciences (Guangzhou, China). A siRNA negative control was included in all experiments as a negative control. Knockdown efficiency was verified by qPCR and WB prior to downstream analyses. In some experiments, the AKT inhibitor MK2206 (10 µm, Selleckchem) and the IL‐1β/IL‐6 inhibitor orientin (Mce, HY‐N0405) were used to suppress the effects of AKT and IL‐1β/IL‐6, respectively.

293T cells (third generation) were kindly provided by Professor Fuyi's laboratory at Capital Medical University and cultured in DMEM supplemented with 10% FBS under 3% CO_2_ at 37 °C. Plasmids expressing Feimin‐WT‐3×FLAG, Feimin‐Mut1‐3×FLAG, Feimin‐Mut2‐3×FLAG, Feimin‐Mut3‐3×FLAG, and AKT‐HA in the pcDNA3.1(+) vector were synthesized and purified by Yunzhou Bio (Guangzhou, China). According to the manufacturer's protocol, each transfection was performed using 2.5 µg of plasmid DNA and Lipofectamine 3000 reagent (Invitrogen, Carlsbad, CA, USA). Cell lysates were collected and analyzed 48 h after transfection.

### Oil Red O Staining

Oil Red O staining was conducted using an Oil Red O Stain Kit (Solarbio, Beijing, China) to assess lipid accumulation in BV‐2 cells. The cells were first fixed with ORO Fixative for 25 min, followed by washing with distilled water and immersion in 60% isopropanol. They were then stained with freshly prepared Oil Red O solution for 15 min. After additional washes with distilled water and ORO buffer, images were acquired using a microscope (Zeiss, Oberkochen, Germany).

### Western Blot

The WB assay was performed as previously described above.^[^ ^33^ ^]^ Briefly, WB analysis was used to quantify the levels of target proteins in BV2 cells. Cells were lysed using RIPA buffer (Solarbio Corporation, Beijing, China) supplemented with protease inhibitors, 1 mM PMSF, and phosphatase inhibitor cocktails (Beyotime Corporation, Shanghai, China). The extracted proteins were separated via 10% SDS‐PAGE and transferred onto polyvinylidene fluoride (PVDF) membranes (Millipore, Sigma Corporation, St. Louis, MO, USA). The membranes were blocked with Tris‐buffered saline containing 0.1% Tween‐20 (TBST) and 5% skim milk. After three washes, the membranes were incubated overnight at 4 °C with primary antibodies, including feimin (1:1000; ThermoFisher, PA5‐113076), AKT (1:1000; Cell Signaling Technology [CST], 4691S), p‐AKT (1:1000; CST, 2965S), mTOR (1:1000; CST, 2972), p‐mTOR (1:1000; CST, 2971), PHLPP1 (ABclonal, A9542), PHLPP2 (ABclonal, A18218), PTEN (ABclonal, A11128), PP2A (ABclonal, A6702), Flag (Proteintech, 20543),Actin (Servicebio, GB15001), GAPDH (ABclonal, A19056). After five washes, the membranes were incubated with horseradish peroxidase (HRP)‐conjugated goat anti‐mouse or goat anti‐rabbit secondary antibodies (Gene‐Protein Link Biological Technology Corporation, Beijing, China) for 1 h. Finally, a chemiluminescent substrate was applied to visualize the target protein bands.

### Immunoprecipitation

The immunoprecipitation (IP)assay was performed as previously described.^[^ ^33^, ^38^ ^]^ Briefly, BV2 were lysed in IP lysis buffer containing NP‐40, 1 mM phenylmethylsulfonyl fluoride (PMSF), and a protease inhibitor cocktail, followed by incubation on ice for 30 min. The lysates were then centrifuged at 12,000 rpm for 20 min at 4 °C to remove debris. Meanwhile, an anti‐AKT antibody was incubated with protein A‐conjugated Dynabeads for 30 min at room temperature (RT) to allow antibody binding. The antibody‐bound dynabeads were then added to the supernatant of the BV2 cell lysate and incubated for 1 h at room temperature with gentle rotation. The immunoprecipitates were washed five times with IP wash buffer to remove nonspecific binding. Finally, the immunoprecipitated complexes were separated using a magnetic rack, and the supernatant was carefully removed. The beads were resuspended in SDS‐PAGE loading buffer and eluted for subsequent WB analysis.

### Construction of Protein‐Protein Interaction Network

To explore the potential interactions between Pi3K, AKT, and mTOR, we conducted an analysis using the Search Tool for the Retrieval of Interacting Genes (STRING) website. The STRING is an online tool used to assess protein‐protein interaction network data.^[^ ^25^ ^]^ Only experimentally validated interactions with a combined score of ≥ 0.4 were considered significant.

### ELISA

Briefly, supernatants were collected from cultured BV2 cells or mouse blood samples. The levels of IL‐1β and IL‐6 in the supernatants were quantified using commercially available ELISA kits (Invitrogen, CA, USA) following the manufacturer's instructions. In summary, capture antibodies were coated onto a 96‐well plate and incubated overnight at 4 °C. Afterward, the plate was washed three times and dried by tapping. The collected supernatants were then added to the antibody‐coated wells and incubated at 37 °C for 30 min. Following another three washes, enzyme‐linked secondary antibodies were added and incubated at 37 °C for 30 min. The plate was then rewashed and dried before adding the substrate solution for color development at 37 °C for 15 min. Finally, the reaction was terminated using a stop solution, and the optical density (OD) was measured using an ELISA detector.

### Immunofluorescence and BODIPY staining of brain and cell sections

Brain and cell sections were subjected to Immunofluorescence (IF) staining using the following primary antibodies: feimin (1:300; ThermoFisher, PA5‐113076), PLIN2 (1:500; ABclonal, A22456), NeuN (1:500; Abcam, ab177487), IBA1 (1:500; Abcam, ab178846), IL‐1β (1:500; Abcam, ab254360) and IL‐6 (1:500; CST, 12912T). Secondary antibodies included Alexa Fluor 488 donkey anti‐goat (1:1000; Thermo Fisher Scientific, A‐11055), Alexa Fluor 488 donkey anti‐rabbit (1:1000; Invitrogen, A‐21206), Alexa Fluor 594 donkey anti‐rabbit (1:1000; Thermo Fisher Scientific, A‐21207), and Alexa Fluor 647 donkey anti‐mouse (1:1000; Invitrogen, A‐31571).

For brain sections, after three washes with phosphate‐buffered saline (PBS; Sigma‐Aldrich, P3813), sections were incubated with 10% donkey serum (Absin, abs935; dissolved in PBS) for 2 h at room temperature (RT). After washing, sections were incubated with primary antibodies, followed by secondary antibodies for 2 h at RT. To visualize LDs in brain tissues, sections were washed and incubated with BODIPY 493/50 solution (1:1000 in PBS; Thermo Fisher Scientific, D3922) for 15 min at RT. Nuclei were counterstained with 4′,6‐diamidino‐2‐phenylindole (DAPI; 1:500; SolelyBio, C0065) for 5 min at RT.

For cell immunofluorescence staining, BV2 cells, and primary microglia seeded onto poly‐l‐lysine‐coated glass coverslips (NEST, 801010) were fixed with 4% PFA for 20  min, washed with PBS, and blocked with 10% donkey serum containing 0.1% Triton X‐100 (Sangon Biotech, A600198) for 2 h at RT. Cells were then incubated with primary antibodies, followed by secondary antibodies for 2 h at RT. BODIPY 493/50 solution and DAPI were used to stain LDs and nuclei, respectively, for 15 min. Sections and cells were imaged at 40× or 63× magnification using confocal scanning laser microscopes (Leica, Frankfurt, Germany; Zeiss, Oberkochen, Germany) with Z‐stack scanning or an automated whole‐slide fluorescence scanner (3DHISTECH, Pannoramic MIDI II), with images visualized using SlideViewer (version 2.5.0). At least five mice per group were included in each experiment. For each section per animal or cell group, five randomly selected fields were imaged for quantitative analysis. According to previous studies,^[^ ^59^, ^60^ ^]^ we performed stereologic quantification of immunofluorescence‐stained sections using systematic random sampling, collecting one section at regular intervals within the region of interest. Image acquisition was conducted under consistent exposure and magnification settings. In ImageJ, all images were batch‐processed using identical threshold parameters for each experiment, and quantification was performed by an investigator blinded to experimental groups. Point counting was used to estimate the proportion of positive areas, and cell counting followed the optical dissector principle, excluding cells touching the exclusion borders. All analyses were conducted under blinded conditions to ensure statistical reliability. The fluorescence intensity was uniformly applied within a fixed gray range of 20–255, and the background threshold was determined using the no‐load control group. Mean fluorescence values were measured and normalized to the control group (set as 1.0) for consistent quantification.

### Hematoxylin and Eosin Staining

Brain tissue samples were fixed with 4% paraformaldehyde, dehydrated using different concentration gradients of ethanol, embedded in paraffin, and cut into sections 4 µm thick. Sections were deparaffinised, stained with hematoxylin and eosin, dehydrated, and mounted with neutral gum. The staining of the tissue sections was imaged under a Nikon microscope (Eclipse, 80i).

### Primary Microglia Cells

For the neonatal mice, primary microglia cells were prepared from cerebral cortices of five to eight mice (1 to 2 days old, mixed sex), feimin^Mic‐/−^ mice, or controls. As previously described,^[^ ^61^, ^62^ ^]^ hippocampus tissues were digested with 0.25% trypsin‐EDTA at 37 °C for 30 min, followed by mechanical trituration in DMEM/F12 supplemented with 10% fetal bovine serum (Gibco, 11320033). The mixed cells were filtered through a 70‐µm strainer and seeded into poly‐D‐lysine–coated T25 flasks (NEST Biotechnology, 707003) with DMEM/F12 containing 1% penicillin‐streptomycin and 10% fetal bovine serum. When confluency was reached after ≈10 days in vitro, the flasks were shaken at 180 rpm for 1 h at 37 °C to detach the microglia loosely adhered to the mixed cell layer. The supernatant containing the detached microglia was collected and reseeded for 1–2 h to allow the microglia to attach. Once attached, cells were gently washed twice with warm 1× PBS to remove aggregates and debris, followed by the addition of fresh culture medium. Microglia were used for experiments when they reached 60–70% confluency.

Primary microglia were isolated as previously described.^[^ ^38^, ^63^ ^]^ Briefly, mice subjected to 12 weeks of HFDs (more than 20 mice per group) were anesthetized with tribromoethanol and perfused with ice‐cold PBS. The hippocampi were carefully dissected and freshly harvested into ice‐cold Hank's Balanced Salt Solution (HBSS). Tissues were digested at 37 °C for 30 min in HBSS containing collagenase II (37.5 U mL^−1^) and DNase I (45 U mL^−1^), with gentle vortexing every 10 min. The resulting cell suspension was passed through a 70‐µm (200 mesh) cell strainer to remove debris and centrifuged at 600 × g for 5 min at 4 °C to collect the cell pellet.

The pellet was resuspended in 37% Percoll solution and subjected to density gradient centrifugation using a three‐layer gradient (70% Percoll, 37% Percoll, and PBS) at 800 × g for 30 min at 4 °C. Cells were collected from the interface between the 37% and 70% layers and washed twice with PBS. Microglia were further purified using CD11b MicroBeads (Precision Biomedicals, 721105) according to the manufacturer's instructions. Finally, purified microglia were collected and used for subsequent experiments.

### Cell Death Analysis

To detect neuronal apoptosis in the HT22 cells and in the brain, TUNEL assay (Beyotime, C1086) was performed on HT22 and brain sections according to the manufacturer's instructions. The stained sections were observed and captured using an inverted fluorescence microscope. The number of TUNEL^+^NeuN^+^ cells was counted. Apoptotic cells were detected using the One Step TUNEL Apoptosis Assay Kit, following the manufacturer's instructions. Samples were briefly incubated with the TUNEL reaction mixture for 1 h at 37 °C, followed by DAPI staining. Apoptotic cells were then visualized and recorded using a confocal fluorescence microscope.

To assess HT22 cell death induced by BV2‐secreted pro‐inflammatory cytokines, the 7‐AAD/Annexin V Apoptosis Detection Kit I (KEYGEN BIOTECH, KGA1026) was used according to the manufacturer's instructions. Briefly, a total of ≥10⁴ cells were resuspended in a binding buffer and incubated with 5 µL of Annexin V‐APC and 5 µL of 7‐AAD in the dark for 15 min. The proportions of cells undergoing early apoptosis and late apoptosis were quantified as the percentage of Annexin V‐APC⁺/7‐AAD^−^ and Annexin V‐APC⁺/7‐AAD⁺ labeled cells, respectively. The stained cells were then analyzed by flow cytometry using a FACSCalibur (BD Biosciences, USA), and data were processed with FlowJo software.

### Cell Viability Assays

Cell viability was assessed using the Cell Counting Kit‐8 (CCK‐8) assay following the manufacturer's instructions (Meilunbio, MA0225). Briefly, 8,000–10,000 cells per well were seeded in 96‐well plates and cultured overnight. The cells were then treated with different concentrations of PA or Orientin. At the indicated time points, 10 µL of CCK‐8 solution was added to 90 µL of culture medium in each well. The cells were incubated for 4 h, and absorbance was measured at 450 nm using a spectrophotometer (TECAN, Switzerland).

### Morris Water Maze

Spatial learning and memory were evaluated using the MWM test, following a previously described protocol.^[^ ^38^, ^64^ ^]^ Briefly, a circular platform (11 cm in diameter) was submerged 1.5 cm below the water surface in the center of one quadrant of a 120‐cm circular pool. The water was made opaque by adding milk powder, and the temperature was maintained at 22° ± 1 °C. Habituation training was conducted on day 0. Learning trials took place over five consecutive days, with four trials per day. In each trial, mice were released from one of four designated positions facing the pool wall and given 60 s to locate the hidden platform. The time taken for each mouse to reach the platform was recorded. On day 6, a probe test was conducted with the platform removed; the number of platform crossings and the residence time in the target quadrant were recorded to assess spatial memory performance. Pharmacokinetic studies confirmed that MK‐2206 readily enters the brain 18 h post‐injection and maintains stable concentrations.^[^ ^65^ ^]^ As described in previous studies, for continued treatment, MK‐2206 was administered orally three times a week for four weeks.^[^ ^65^ ^]^ Behavioral scoring was performed by investigators blinded to group allocation. All animals that showed signs of motor defects or diseases in behavioral experiments were excluded. All investigators involved in behavioral scoring and histological analyses were blinded to group allocation.

### Novel Object Recognition Test

The habituation, familiarization, and recognition memory tests were conducted as previously described.^[^ ^66^ ^]^ For the habituation trial, a repeated procedure was performed over three consecutive days, during which mice were allowed to freely explore an empty open‐field box for 5 min each day. In the familiarization session on the following day, each mouse was placed in the same open‐field box containing two identical objects for 10 min. The recognition memory test was conducted 24 h later in the same box, with one familiar object replaced by a novel object that differed in shape, color, and texture. Mice were given 10 min to explore, and their exploration and sniffing time for each object was recorded via video analysis. Exploration was defined as the mouse orienting its nose toward the object at ≤2 cm. Recognition memory was assessed using the discrimination index, calculated as the ratio of time spent exploring the novel object to the total time spent exploring both the novel and familiar objects.

### Y‐Maze Spontaneous Alternation Test

As described in the previous study,^[^ ^33^ ^]^ short‐term spatial memory performance was assessed using the spontaneous alternation test. Mice were gently placed in the starting arm (each arm 35 cm long and 5 cm wide, with 15 cm high walls) and allowed to explore freely for a single 8‐min trial. During this period, they could navigate through all three arms of the Y‐maze. The movement paths were recorded using a digital video recorder, and alternation behavior was analyzed with SMART 3.0 software (Pan‐lab). A successful alternation was defined as consecutive entries into all three arms in sequence. The alternation rate was calculated as the number of successful alternations divided by the total number of arm entries minus two.

### Food Intake

Evaluating dietary consumption was based on previous literature,^[^ ^67^ ^]^ and food intake was measured under HFD conditions. The mice adapted in the cage for three days in advance. A pre‐weighed amount of HFD pellets was provided at the start of each 24 h, and the remaining food was carefully collected and weighed. Daily intake per cage was calculated as the difference in food weight, and the average intake per mouse was estimated by dividing by the number of animals per cage (n = 10 mice, n  =  4 biological replicates). Measurements were conducted over 2 consecutive days to obtain stable averages. All cages were maintained under identical environmental and feeding conditions to minimize variability.

### Tissue Perfusion and Collection

After cognitive performance assessment, mice were anesthetized and intracardially perfused with 0.9% sodium chloride (Sangon Biotech, A610476), followed by 4% paraformaldehyde (PFA; Sigma‐Aldrich, 158127). Whole brains were harvested via decapitation, fixed in 4% PFA for 12 h. Samples were then dehydrated in gradient sucrose solutions (Sangon Biotech, A610498) at 4 °C, and sectioned at 40 µm for immunofluorescence, Oil Red O staining, and TUNEL assays. For qPCR analysis, fresh bilateral hippocampal tissues were collected post‐anesthesia and stored at −80 °C for subsequent RNA isolation and total protein extraction. For plasma collection, blood samples were obtained from the orbital plexus after anesthesia. EDTA plasma was isolated by centrifugation as described above and stored at −80 °C for further analysis. For each specified experiment, at least five mice per group were sacrificed for brain or plasma collection.

### Statistical Analysis and Reproducibility

Statistical analysis was conducted using GraphPad Prism 9.0 software (GraphPad Software Inc., San Diego, CA, USA). An independent samples t‐test was used for continuous variables with a normal distribution, while the Mann–Whitney U‐test was applied for non‐normally distributed continuous variables. When comparing among multiple groups, we used one‐way ANOVA and conducted Tukey's post hoc test as needed. Bonferroni post hoc correction for multiple pairwise comparisons within each experimental group. Data are presented as mean ± SD. All experiments were randomized, and investigators responsible for data collection were blinded to imaging and data analysis. No statistical methods were used to predetermine sample sizes; instead, our sample sizes are similar to or larger than those reported in prior studies.^[^ ^33^, ^68^ ^]^ Detailed information on sample size, number of replicates, and statistical tests used for each experiment is provided in the figure legends. A two‐sided *p*‐value < 0.05 was considered statistically significant.

### Reporting summary

Further information on research design is available in the Nature Portfolio Reporting Summary linked to this article.

## Conflict of interests

The authors declare that there are no conflicts of interest.

## Author contributions

Renxi Wang and Ran Gao conceived and initiated the project: Ran Gao and Zhonghua Xiong performed the experiments, analyzed the data, and wrote the manuscript. Renxi Wang revised the manuscript. All authors contributed to the interpretation of the results, critical revision of the manuscript, and approved the final version of the manuscript. All authors read and approved the final manuscript.

## Ethics statement

The study was approved by the Research Ethics Committee at Capital Medical University (Approval code: AEEI‐2020‐187. Approval date: 1 December 2020).

## Supporting information



Supporting Information

## Data Availability

The data that support the findings of this study are available from the corresponding author upon reasonable request.
