# Supplementary material for: Microglial Feimin Alleviates Cognitive Impairment in High‐Fat Diet‐Fed Mice
Source: Adv Sci (Weinh). 2025 Oct 20;12(48):e12023. doi: 10.1002/advs.202512023 (PMC12752604; doi:10.1002/advs.202512023)
Supplement: Supplementary file 1 — Supporting Information [file ADVS-12-e12023-s001.docx]

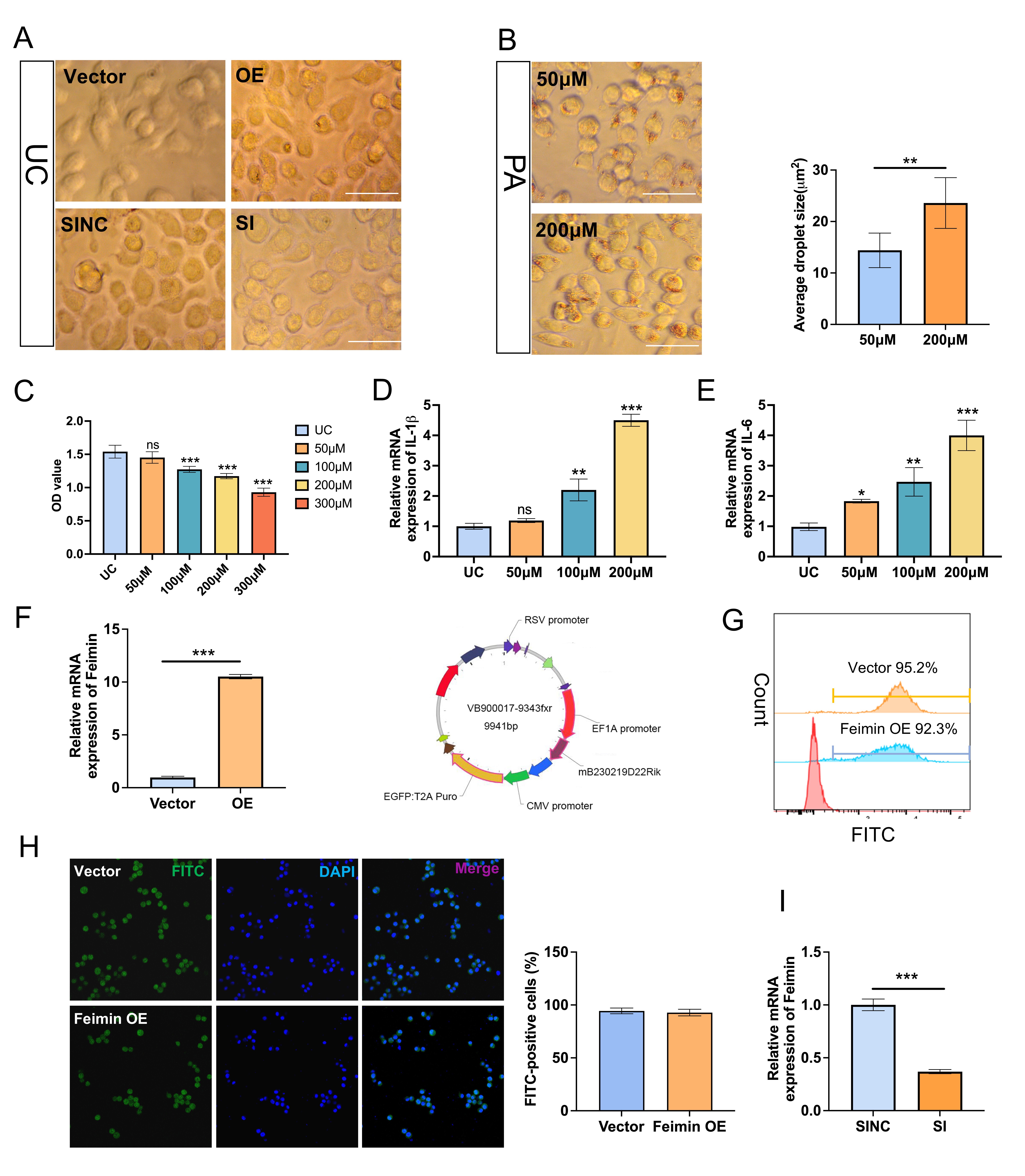


Sup Fig 1: PA Concentration Gradient Validation. Related to Figure 1. (A) Under basal conditions, no lipid droplets were detected in vector control, feimin OE, control siRNA, or feimin siRNA cells (n = 5, *p* > 0.05). (B) BV2 cells were cultured for 24 hours in serum-free medium containing different concentrations of PA and quantification of lipid droplet accumulation are shown. (n = 5, *p* = 0.005) (C) BV2 cells were incubated in serum-free medium with varying concentrations of PA for 24 h, and cell viability was assessed by CCK-8 assay (n = 5, *p* = 0.0005,0.0004, and 0.0007). (D-E) BV2 cells were incubated for 24 h in serum-free medium with increasing PA concentrations, and (D) IL-1β (*p* > 0.05, *p* = 0.003, 0.0001) and (E) IL-6 (*p* > 0.05, *p* = 0.02, 0.001,and 0.0004) mRNA levels were measured by qPCR (n = 3, **p*< 0.05, ***p* < 0.01, ****p* < 0.001). (F) BV2 cells were stably transfected with feimin OE lentivirus, and successful overexpression of feimin mRNA was validated by qPCR. N = 3, *p* = 0. 0004.The schematic of the lentiviral construct is included. (G) BV2 cells without fluorescent labeling were used as a negative control, BV2 cells transduced with either empty vector or feimin-OE lentivirus were stained with FITC-conjugated antibodies, and transduction efficiency was assessed by flow cytometry, n = 3. (H) BV2 cells were transduced with either an empty vector or a feimin-OE lentivirus, and FITC-positive cells were visualized by immunofluorescence microscopy as shown in the schematic diagram. Quantification of FITC-positive cells (%). Scale bars: 50 μm (I) Feimin mRNA expression in BV2 cells transfected with feimin siRNA was analyzed by qPCR. N = 3, *p* = 0.0004. Data were presented as means ± SD of three independent experiments. (B, F, I)) Two-tail Student's T-test, (C-E) One-Way ANOVA.


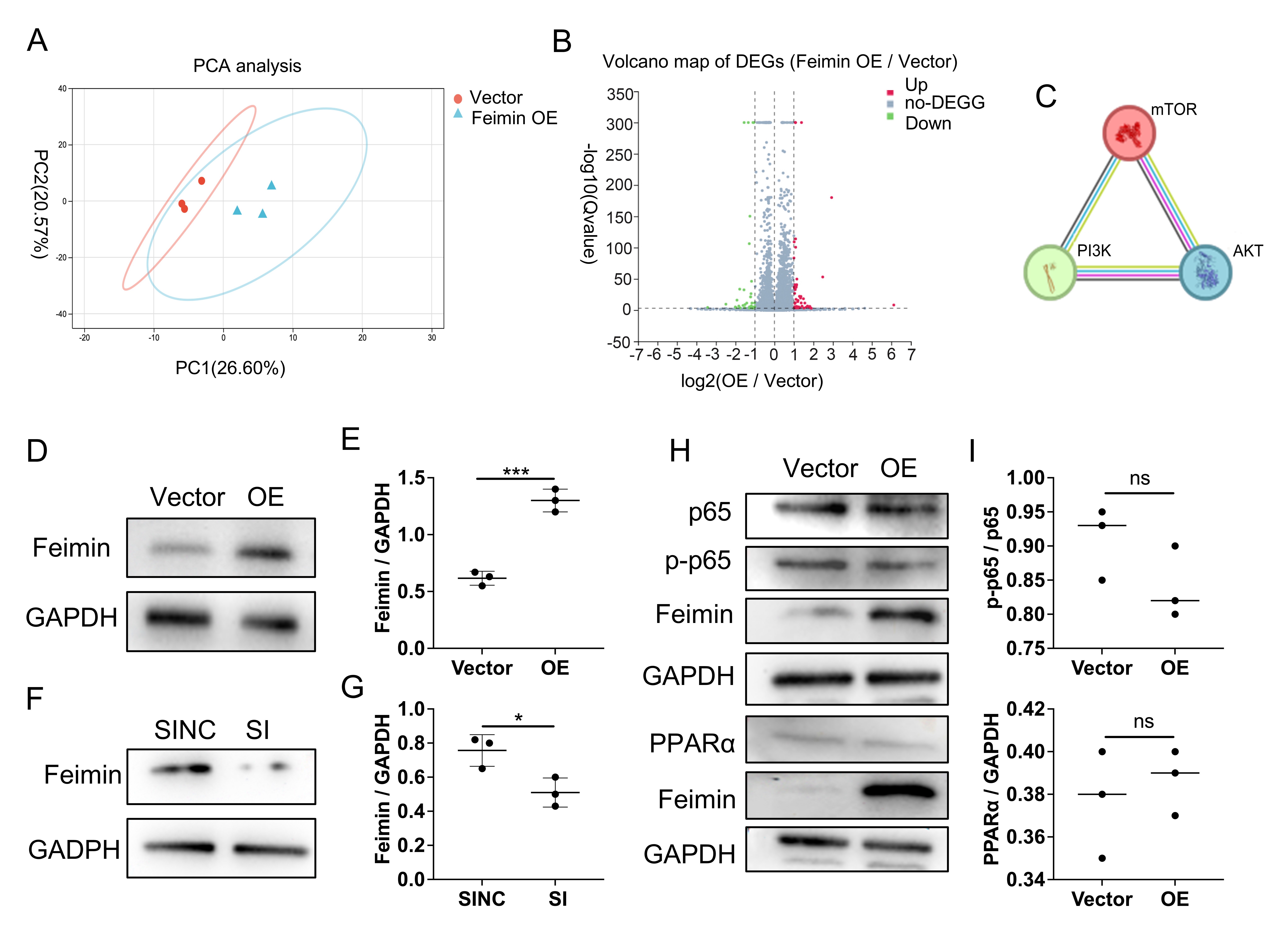


Sup Fig 2: Related to Figure 2. (A, B) The standard QC diagrams (PCA, volcano diagrams) of KEGG (Vector and feimin OE groups, with n=3 in each group). (C) PPI network. The PPI network of PI3K, AKT, and mTOR. The red line indicates that the two have been experimentally determined. (D-E). Overexpression of feimin in BV2 cells. N = 3, *p* = 0.0003. (F-G) Knockdown of feimin in BV2 cells. N = 3, *p* = 0.042. (H-I) WB analysis and quantification of p-p65, and PPARα expression in BV2 transduced with control vector or feimin OE, n = 3, *p* > 0.05. (C, D, F) Data were presented as means ± SD of three independent experiments. (C, D, G)) Two-tail Student's T-test.





Sup Fig 3: Mouse feimin conditional knockout project. Related to Figure 3. (A) An overview of target strategies in mice. (B) Dot Plot and GC Content Overview with BLAT Results. Dot plot self-alignment revealed no significant tandem repeats. GC content analysis showed no regions of high GC density. BLAT search of the 3 kb regions upstream and downstream of Exon 2 identified no significant off-target similarity in the genome. (C) Identification results of three genotypes of mice tails. (D-F) Results of feimin WB and mRNA level in feimin^Mic-/-^ adult mice primary microglia compared with feimin^fl/fl^ adult mice primary microglia (n = 3, *p* = 0.0032 and 0.0045). (G-H) After 24 hours of PA treatment, neonatal primary microglia (feimin^Mic−/−^) were pretreated with DMSO or MK2206 (an AKT inhibitor, 10 μM) for 30 minutes. Cells were then immunostained for PLIN2, BODIPY (to label LDs) (n=5, *p* = 0.0043 and 0.0005). (I-J) Nascent feimin^Mic−/−^ primary microglia were pretreated with DMSO or MK2206 for 30 minutes, followed by PA treatment for 24 hours. IL-1β and IL-6 mRNA levels were measured by qPCR (n = 5, *p* = 0.0003, *****p* < 0.0001). (E-F) Two-tail Student's T-test, data were presented as means ± SD of three independent experiments.


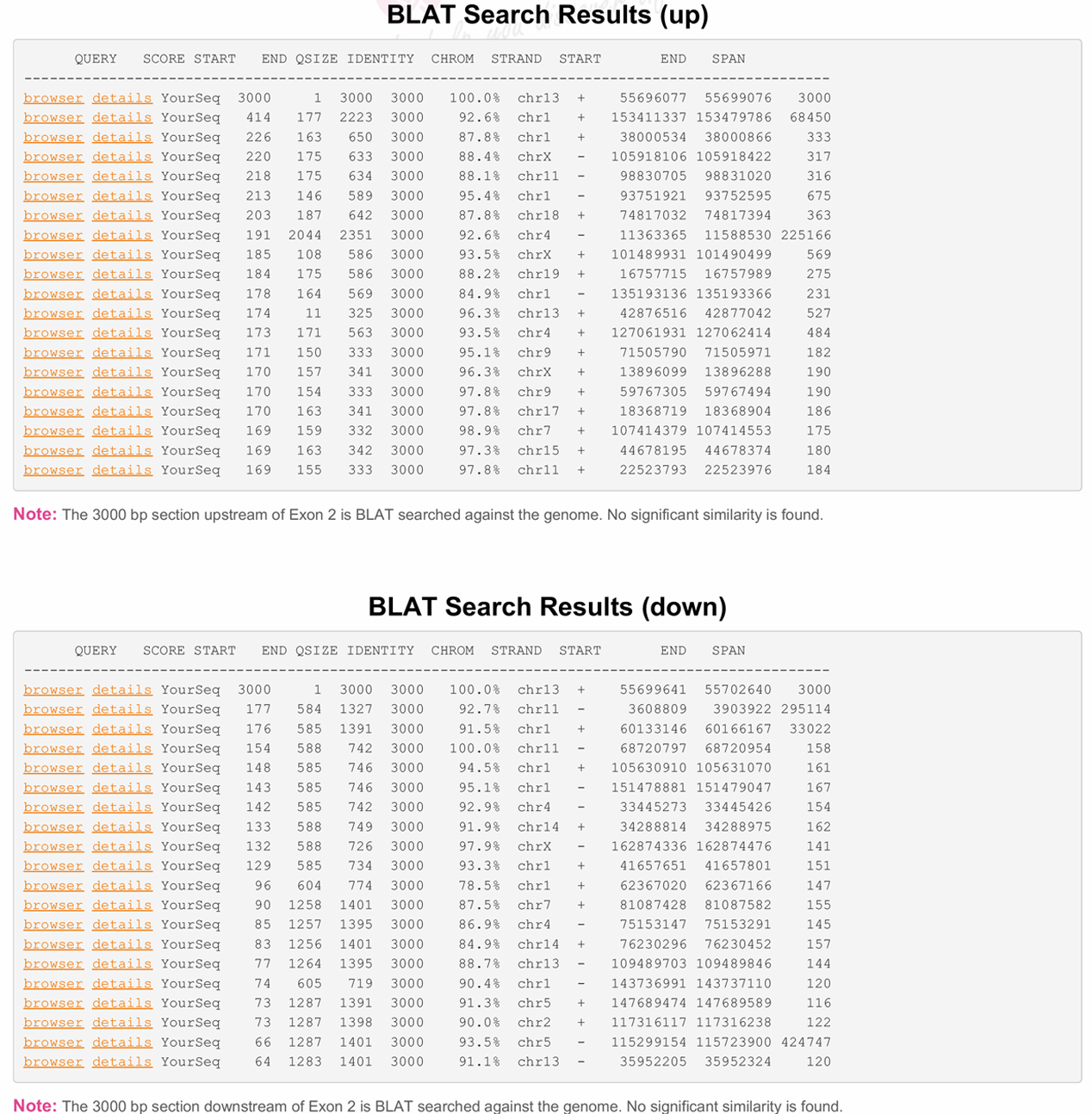


Sup Fig 4: BLAT search of the 3 kb regions upstream and downstream of Exon 2 identified no significant off-target similarity in the genome.


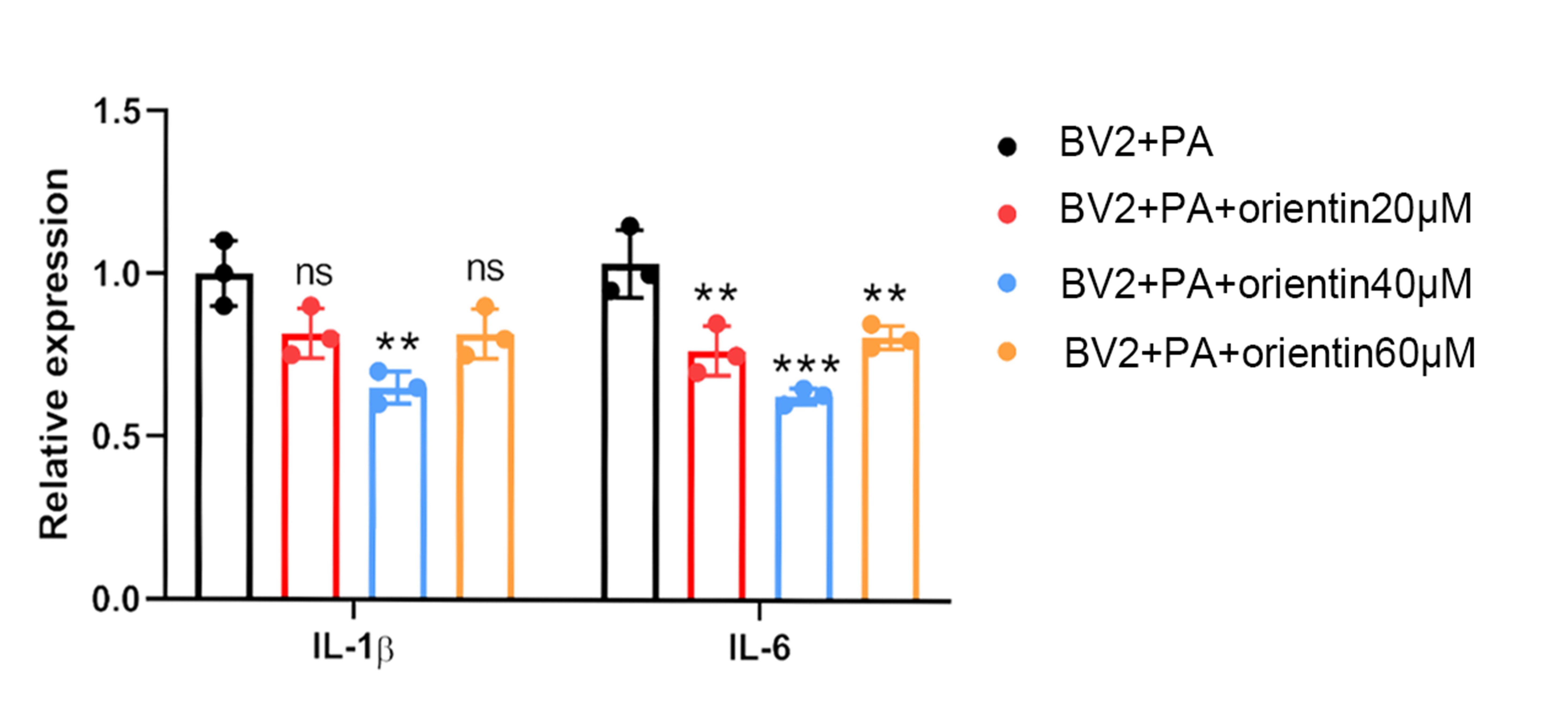


Sup Fig 5: Validation of the inhibitory effect of Orientin on IL-1β and IL-6. Related to Figure 4. BV2 cells were stimulated with PA and treated with Orientin at concentrations of 20 µM, 40 µM, and 60 µM, respectively, followed by qPCR analysis to assess IL-1β and IL-6 mRNA expression (n = 3, one-Way ANOVA, data were presented as means ± SD of three independent experiments, ***p* < 0.01, ****p* < 0.001, *****p* < 0.0001).


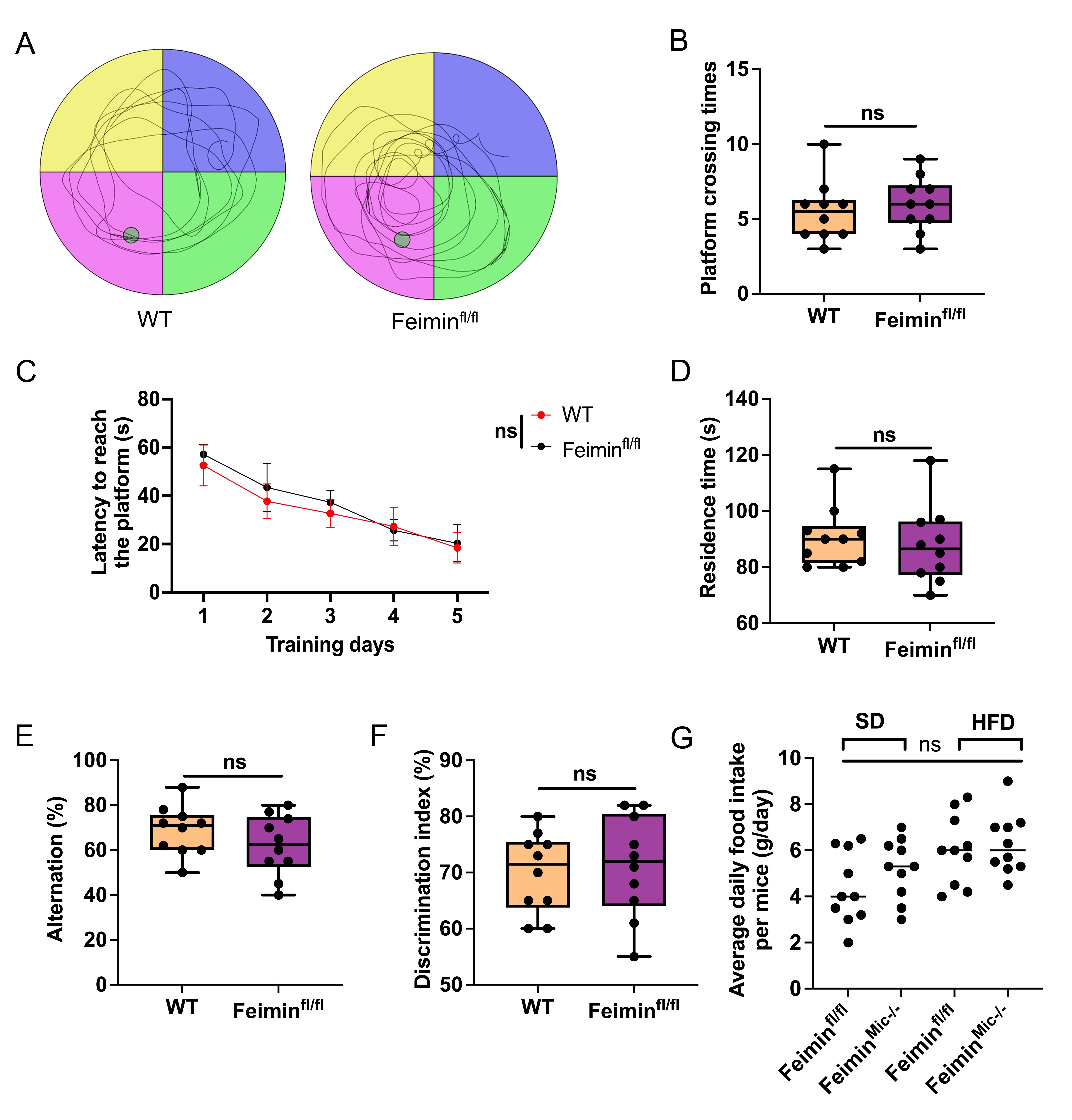


Sup Fig 6: Cognitive behavioral performance of WT and feimin^fl/fl^ mice. Related to Figure 5. (A) Representative positioning and navigation tracks of the Morris Water Maze (MWM) test in the WT and feimin^fl/fl^ mice groups. (B) The platform crossing times, (C) time to reach the platform during the training period, and (D) time spent in the target quadrant in MWM tests were shown. (E) Alternation rate in the Y-maze test. (F) Discrimination index. In each test, n = 9 mice. (G) Daily food intake was measured over the course of SD and HFD. No significant difference was observed between feimin^fl/fl^ and feimin^Mic−/−^ mice (n = 10 per group, *p* > 0.05). WT: wild type. Data were presented as means ± SD of three independent experiments. (B-F) Unpaired two-tailed Student’s t-test, (G) one-Way ANOVA, *p* >0.05.


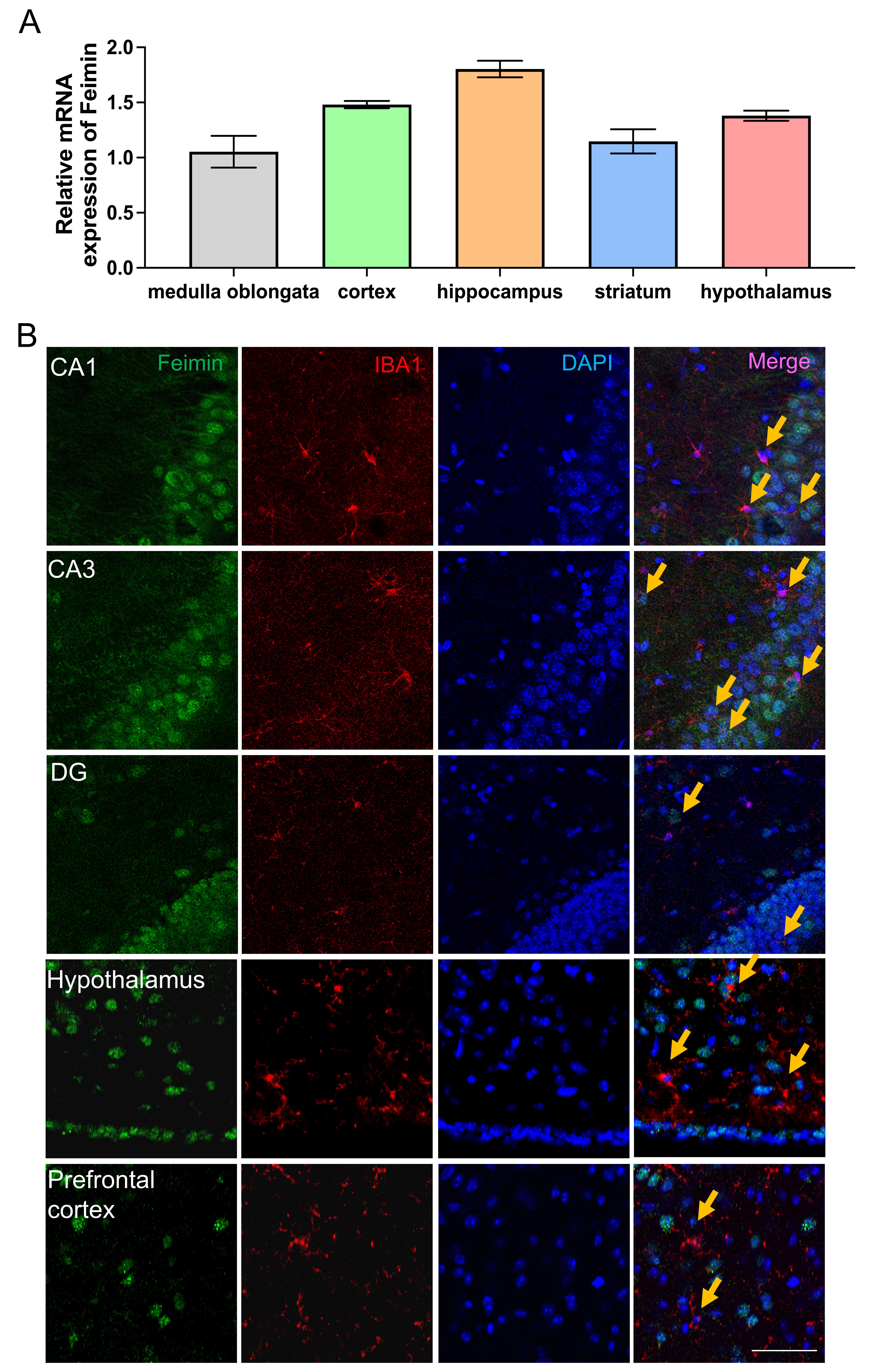


Sup Fig 7: The main distribution of feimin in brain region. Related to Figure 6. (A) The expression levels of feimin mRNA in different brain regions in WT mice. (B)The expression of feimin in brain sections of WT mice. Representative micrographs of feimin and IBA1^+^ (microglia) in the brain sections of WT mice. (n = 5 mice per group) Scale bar: 50 μm.


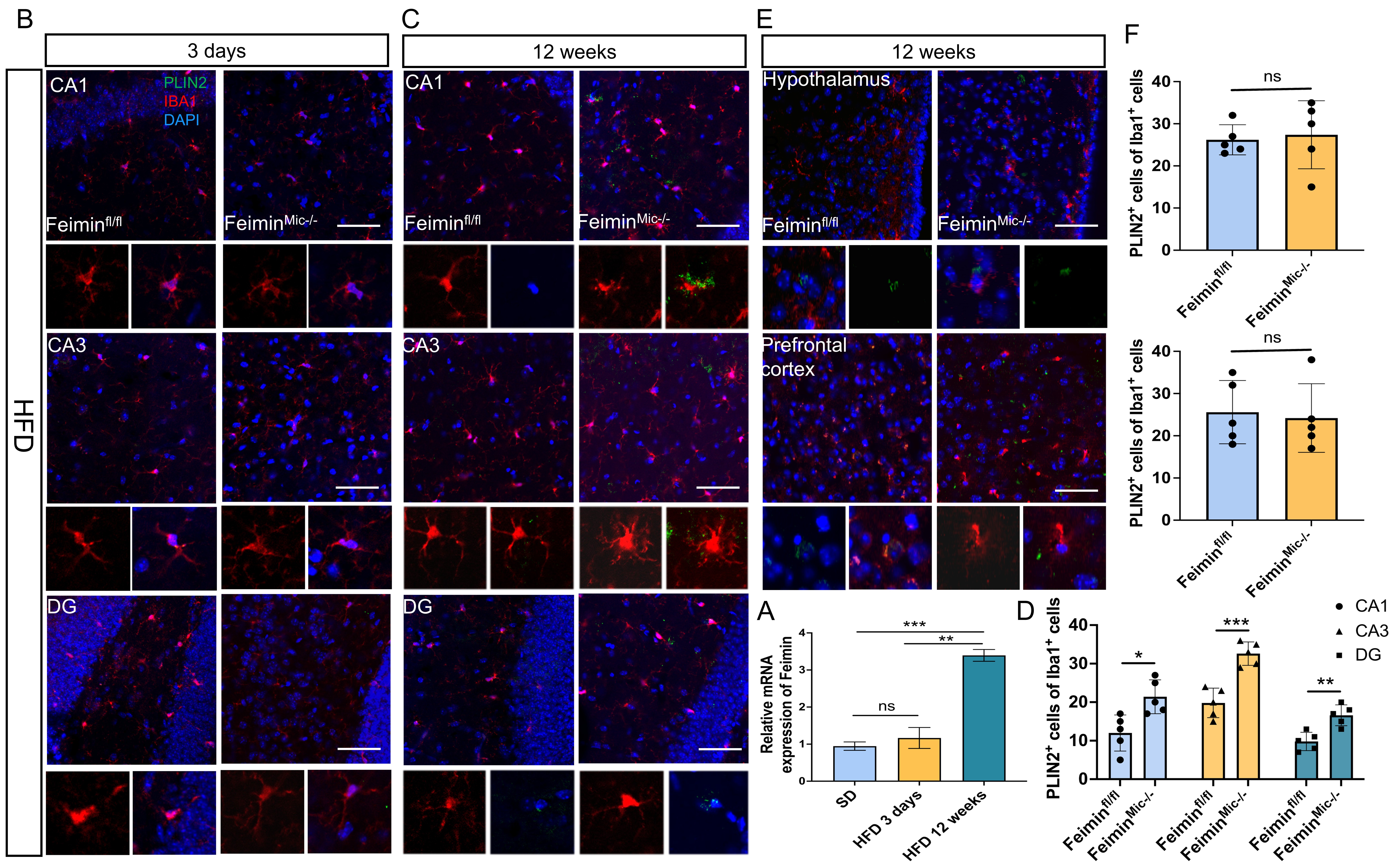


Sup Fig 8: Increased LD accumulation in the hippocampus of feimin^Mic-/-^ models under HFD. Related to Figure 6. (A) Relative mRNA expression of feimin in the hippocampus under multiple time points (n = 5, *p* = 0.0003). (B, C) Immunostaining of PLIN2^+^ (LDs) and IBA1^+^ (microglia), and quantification of PLIN2+ microglia in the hippocampi of feimin^fl/fl^ and feimin^Mic-/-^ mice in the HFD 3 days and 12 weeks model. (D) Brain tissues analysis after 12 weeks of HFD was performed across three independent experiments (n = 5, *p* = 0.011,0.0003 and 0.004, respectively). (E, F) Immunostaining of PLIN2⁺ lipid droplets and IBA1⁺ microglia in the hypothalamus and prefrontal cortex of feimin^fl/fl^ and feimin^Mic-/-^ mice in the 12-week HFD model, with quantification of PLIN2⁺ microglia. Brain tissue analysis was conducted across three independent experiments (n = 5 mice per group, *p* > 0.05). Scale bar: 50 μm. Unpaired two-tailed Student’s t-test, data were presented as means ± SD of three independent experiments.





Sup Fig 9: No statistically significant difference in inflammatory factors among SD mice. Related to Figure 7. (A) HE staining in both feimin^fl/fl^ and feimin^Mic−/−^ groups under SD conditions. No significant difference was found in the DG region, scale bar: 50 μm, n = 5 per group, *p* > 0.05. (B) Representative micrographs of IL-1β or IL-6 with IBA1 in the CA1, CA3, and DG regions of mice from feimin^fl/fl^ and feimin^Mic-/-^ mice groups. (C) The numbers of IL-1β^+^, IL-6^+^ of IBA1^+^cells were quantified (n = 6, *p* > 0.05). (D-E) Statistics of the number of IBA1^+^cells, sup Fig. 8D displays the corresponding fluorescence distributions from Figure 7B, C. n = 6 per group, scale bar: 50 μm. Brain tissues were analyzed from three independent experiments (n = 6, *p* = 0.03 and 0.001).(F, G) Immunofluorescence staining of IL-1 or IL-6 and IBA1 in the CA3 region of feimin^fl/fl^ and feimin^Mic-/-^ mice after 3 days of HFD treatment, along with a timeline showing the percentage of IBA1⁺ microglia expressing IL-1 or IL-6 in different groups. (n = 6) (C) One-Way ANOVA, (A, E, G) Unpaired two-tailed Student’s t-test, **p* < 0.05, ***p* < 0.01, ****p* < 0.001.

Sup Table 1. Primer sequences in RT-qPCR.

| Gene | Sequence |
| --- | --- |
| B23 | F: 5’- ACGAGTGGTGAAGTCAAGCC -3’ |
|  | R: 5’- CAGAAACAGCACTGGGGAGT -3’ |
| IL-1β | F: 5’-CATTGCTCAAGTGTCTGAAGC-3’ |
|  | R: 5’-GGTCGGAGATTCGTAGCTG-3’ |
| IL-6 | F: 5’-GTACATCCTCGACGGCATCTC-3’ |
|  | R: 5’-GCTCTGGCTTGTTCCTCACTAC-3’ |
| GAPDH | F: 5’-GTCCACTGGCGTCTTCACC-3’ |
|  | R: 5’-ATGAGTCCTTCCACGATACCAA-3’ |

Note: F, forward; R, reverse.
